# Fast and scalable search of whole-slide images via self-supervised deep learning

**DOI:** 10.1038/s41551-022-00929-8

**Published:** 2022-10-10

**Authors:** Chengkuan Chen, Ming Y. Lu, Drew F. K. Williamson, Tiffany Y. Chen, Andrew J. Schaumberg, Faisal Mahmood

**Affiliations:** 1 Department of Pathology, Brigham and Women’s Hospital, Harvard Medical School, Boston, MA USA; 2 Department of Pathology, Massachusetts General Hospital, Harvard Medical School, Boston, MA USA; 3 Cancer Program, Broad Institute of Harvard and MIT, Cambridge, MA USA; 4 Cancer Data Science Program, Dana-Farber Cancer Institute, Boston, MA USA; 5 Department of Electrical Engineering and Computer Science, Massachusetts Institute of Technology, Cambridge, MA USA; 6 Harvard Data Science Initiative, Harvard University, Cambridge, MA USA

**Keywords:** Bioinformatics, Medical imaging, Biomedical engineering, Pathology

## Abstract

The adoption of digital pathology has enabled the curation of large repositories of gigapixel whole-slide images (WSIs). Computationally identifying WSIs with similar morphologic features within large repositories without requiring supervised training can have significant applications. However, the retrieval speeds of algorithms for searching similar WSIs often scale with the repository size, which limits their clinical and research potential. Here we show that self-supervised deep learning can be leveraged to search for and retrieve WSIs at speeds that are independent of repository size. The algorithm, which we named SISH (for self-supervised image search for histology) and provide as an open-source package, requires only slide-level annotations for training, encodes WSIs into meaningful discrete latent representations and leverages a tree data structure for fast searching followed by an uncertainty-based ranking algorithm for WSI retrieval. We evaluated SISH on multiple tasks (including retrieval tasks based on tissue-patch queries) and on datasets spanning over 22,000 patient cases and 56 disease subtypes. SISH can also be used to aid the diagnosis of rare cancer types for which the number of available WSIs is often insufficient to train supervised deep-learning models.

## Abstract (web summary)

A self-supervised deep-learning algorithm searches for and retrieves gigapixel whole-slide images at speeds that are independent of the size of the image repository

## Main

The increasing availability of technologies allowing for the routine creation of high-resolution whole-slide images (WSIs) has triggered tremendous excitement for the field of digital pathology. Whereas the rich morphologic content analysed by pathologists was once locked in glass slides, whole-slide imaging systems now allow pathologists and researchers to access that data digitally without a microscope at hand. Studies demonstrating non-inferiority of WSIs^1–3^ and Food and Drug Administration (FDA) approvals for primary diagnosis to be performed on WSIs mean that pathologists can now adopt these systems for clinical use. However, as institutions scan and store an increasing number of images, they often turn to WSI storage and retrieval paradigms identical to that used for their glass slides—large repositories of data searchable through patient identifiers, case number, date of procedure, pathology report and so on, without leveraging the digital morphologic content of the images themselves.

Meanwhile, the revolution of artificial intelligence^4,5^ (for example, deep learning) in recent years has shown potential in various tasks in pathology that range from disease diagnosis, prognosis and integrative multi-omic analysis^6–17^. However, a majority of computational pathology methods are based on supervised deep learning using slide or case level labels to tackle classification or ranking problems. By comparison, an image search tool that harnesses the rich, spatially resolved information in pathology images is much more powerful for a variety of different applications. For example, finding cases with similar morphologic features can assist in diagnosing rare diseases and unusual conditions that may not have enough cases available for accurate supervised classification models to be developed. Other examples include finding cases with similar morphologies to predict outcome for clinical trials with limited samples, identifying similar cases for teaching and parsing large historical repositories in the absence of electronic pathology reports. A critical challenge that hinders large scale, efficient adoption of histology whole-slide image search and retrieval systems is scalability. This is a unique challenge for WSI retrieval systems^18^ as compared with other image databases since they need to efficiently search a growing number of slides that can each consist of billions of pixels and be several gigabytes in size.

Due to the computationally prohibitive size of WSIs, most approaches split them into smaller image patches and either focus on patch or region of interest (ROI) retrieval that is tailored to specific applications^19–33^. These implementations often need expert pathologists to exhaustively delineate the ROIs, making the system difficult to scale. Recent work has demonstrated promising patch retrieval results without using manual labels by comparing patches in a continuous embedding space using an encoder pretrained by deep metric learning on a large cohort of natural images^34^. However, this approach is limited to small image patches, required considerable computing resources and had a search speed that scaled with the size of the database. Recent work has also shown that representative image patches, embedded into binarized features using encoders pre-trained on real world images, can be used for WSI level retrieval^35,36^. However, the disadvantage of this method is slow search speed on larger datasets due to $O(n\log (n))$ computational complexity, where *n* is the number of WSIs in the database. Additionally, the reported performance degrades when the distribution of the numbers of slides is skewed towards a subset of anatomical sites, which is commonly seen in real-world histology datasets. Other recent studies propose improved feature representation for WSIs by creating permutation invariant embeddings^37^ or fine-tuning pretrained networks on data with morphological information^38^. Scalibility to large histological datasets that capture real-world imbalances in disease types is crucial for a practical and broadly applicable search engine for histology.

Here we propose self-supervised image search for histology (SISH) as a search pipeline that addresses the issues summarized above. SISH theoretically achieves constant time query speed by representing a WSI as a set of integers and binary codes, and does not require any pixel or ROI level annotations. We evaluate SISH on several tasks: first, performance on disease subtype retrieval from a fixed anatomic site is assessed on three cohorts of data, specifically, the primary diagnostic slides in The Cancer Genome Atlas (TCGA)^39^, the Clinical Proteomic Tumour Analysis Consortium (CPTAC)^40^ and slides digitized in-house at the Brigham and Women’s Hospital (BWH). Second, performance on retrieving slides from the same anatomic site as the query, which we evaluate using the TCGA dataset. In total, we used 22,385 diagnostic whole-slide images across 13 anatomic sites and 56 disease subtypes. Third, we demonstrate the utility of SISH for diagnosis of rare cancer types using in-house and TCGA data. Fourth, we show that SISH can be used for patch-level similar morphologic feature search on a variety of different disease models.

**Fig. 1:**
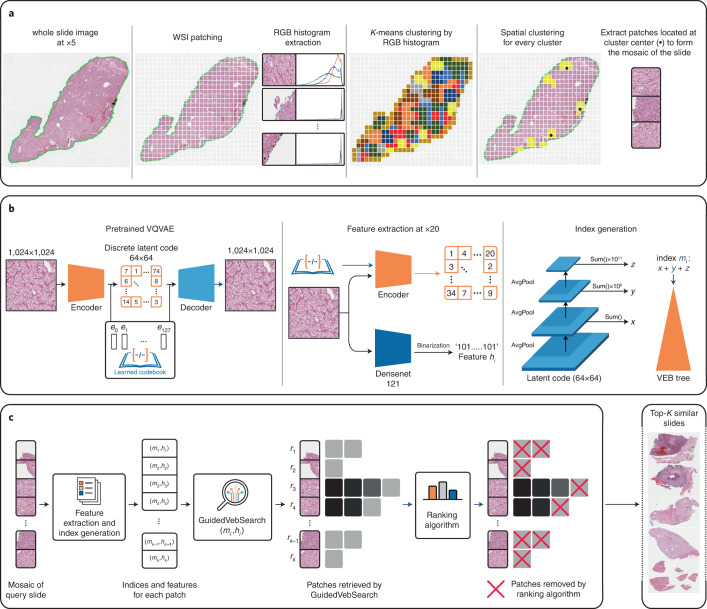
Overview of the SISH pipeline. **a**, After tissue segmentation, we tile the foreground regions and perform two-stage *K*-means clustering to select representative patches to include in the WSI mosaic. We first cluster all patches based on their RGB histogram features. In each cluster generated from the first stage (for example, the yellow cluster shown in the figure), we perform *K*-means clustering again using the spatial coordinates of each patch as features (spatial clustering), extract the patches that correspond to the coordinates of each resulting cluster centre (black dots) and add them to the mosaic of the slide. **b**, We pretrain a VQ-VAE on tissue patches from slides in the TCGA and save its encoder and codebook for feature extraction. For each patch in the mosaic, the VQ-VAE encoder is used to compute its discrete latent representation and a Densenet121 encoder is used to obtain a binarized texture feature vector. Finally, we feed the discrete latent representation into another pipeline composed of a series of average pooling (AvgPool), shift and summation operations to get an integer index for the patch, then use the vEB tree to construct the index structure for search. **c**, For a given query slide preprocessed as a mosaic representation, we feed the mosaic into the feature extractor to compute the integer indices and binarized texture features of each patch in the mosaic, then apply our search and ranking algorithm to filter the candidate patches. See Fig. 2 for more details.

**Fig. 2:**
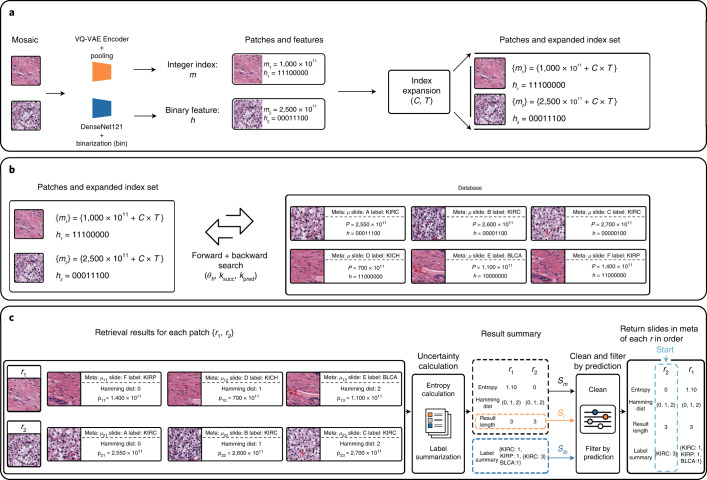
Detailed illustration of search. **a**, Starting from the mosaic of a WSI where a patch could contain normal tissue or morphology of a cancer subtype, SISH encodes each patch into both an integer and binary string representation, using a VQ-VAE encoder and a DenseNet121 encoder pretrained on ImageNet respectively. The pooling operation consists of a series of average pool, summation and multiplication explained in Methods. The binarization process converts a continuous vector to a binary string by starting from *∞*, then walking through all elements in the vector to compare the value of the current element to that of the next one. If the next value is smaller than the current, it assigns the current value to 0, and 1 otherwise. Afterwards, for each patch in the mosaic, SISH expands its index into a set of candidate indices. *C* and *T* are hyperparameters used during expansion (see Guided search algorithm section of Methods). **b**, For each patch, SISH applies the search function to each index in the set of candidate indices. The search function returns the patches within *k*_succ_ successors and *k*_pred_ predecessors in the database whose Hamming distance from the patch in the query mosaic is smaller than *θ*_*h*_. Each patch in the database is associated with an index *p* and metadata *μ* defined in Methods. **c**, For each result *r*, SISH calculates its entropy (by considering the frequency of occurrence of slide labels) and returns summary variables *S*_*m*_, *S*_*l*_ and *S*_*lb*_ for cleaning. In the example shown in the figure, the cleaning function removes outliers in {*r*_1_, *r*_2_}. A result *r* is considered as an outlier if its length (number of patches retrieved) is greater than the *O*_*h*_ or smaller than the *O*_*l*_ percentile. At the same time, the function also removes patches within each *r* whose Hamming distance is greater than the average of the top-*k* in *r*. Lastly, SISH takes a majority vote of the top-5 slide labels within each *r* to remove patches whose slide labels disagree with the majority vote and extract the slides from the *r* with the lowest entropy (see the corresponding sections in Methods for details of entropy calculation, clean and filter-by-prediction).

SISH theoretically achieves *O*(1) constant speed complexity for search, insertion and deletion operations (that is, the speed of the operations does not depend on the size of the database) and supports both slide and patch-level retrieval. SISH leverages a set-based representation of WSI which has better transparency and does not need further supervised training as compared to a continuous vector representation^37^. Specifically, we sample a subset of representative patches (termed a ‘mosaic’) for each WSI by clustering at low resolution to address the gigapixel size of WSIs. SISH uses a Vector Quantized-Variational AutoEncoder (VQ-VAE)^41^ trained on a large dataset in a self-supervised manner and leverages the learned, discrete latent codes to create integer indices for patches in a WSI mosaic. VQ-VAE is a self-supervised approach that learns to generate a small number of descriptive latent codes for each input object. With the integer representation of a slide, we can benefit from the O(loglog(*M*)) search, insertion and deletion speed for integers whose values are within the range [0, *M*], provided by the Van Emde Boas tree (vEB tree)^42^, where *M* is a fixed constant in our pipeline (see Methods for additional details). Our approach does not require comparing the regions of the query WSI against regions of every other WSI in the database: we use the vEB tree to first identify a constant number of potential candidates for each patch in the query mosaic and then use a ranking module to identify the most promising patches that are useful for retrieval (see Supplementary Table 17). These patches often contain meaningful ROIs, and they can be visualized by the human user to provide model interpretability, which is desirable in medical applications to enable more transparent and informed decision-making. Finally, we make the source code of SISH open access (https://github.com/mahmoodlab/SISH) for future studies. An overview of the SISH pipeline is shown in Fig. 1, and the detailed search process is illustrated in Fig. 2.

SISH begins by distilling a mosaic representation of a given slide^36^ (Fig. 1a). To select the patches used for representing the slide, we use two-stage *K*-means clustering. Specifically, we first apply *K*-means clustering on the red, green and blue (RGB) histogram features extracted from patches at ×5 magnification, followed by *K*-means clustering on the coordinates of patches at ×20 magnification within each initial cluster. We extract image patches corresponding the coordinates of the set of final cluster centres and use them as a mosaic representation of the given slide. To convert the patches into a set of integers and binary codes (Fig. 1b), we train a VQ-VAE, which is a variant of the Variational Autoencoder^43^ that gives the input a discrete latent code from a codebook learned on the TCGA slides at ×20 scanner magnification (×200 effective magnification). The codebook generated from the VQ-VAE is held constant throughout all experiments in our study and is not retrained on any of the independent datasets. We use the encoder of the pretrained VQ-VAE along with the learned codebook to encode the patches at ×20 magnification and extract patch features by using a Densenet^44^ model and a binarization algorithm. The last step is to convert the discrete latent codes into integers to store the mosaics in the vEB tree. We feed the latent codes of the mosaics into a pipeline composed of a series of average pooling (AvgPools), summation and shift operations. The intuition behind this pipeline is to summarize the information in each scale via summation, then store it into a different range of digits in an integer.

During search (Figs. 1c and 2), we extract the features of the preprocessed mosaic of the query whole-slide image and then apply the proposed guided search algorithm (GSA) to find the most similar results of each query mosaic. The design principle of GSA is to find a fixed number of nearest neighbours using the vEB and only select the neighbours whose Hamming distances from the patches in the query mosaic are below a certain threshold *θ*_*h*_. The search result of each patch in the mosaic is a list of patches. Each patch contains metadata that document the name of the slide where the patch is located, the diagnosis of the slide and the Hamming distance between the patch in the database and that in the query mosaic. Once each patch in the query mosaic gets its search results, our ranking algorithm ranks the candidate patches used to retrieve the final top-*K* similar slides. We collect all slides that appear in the search results from the candidate patches and sort them on the basis of Hamming distance in ascending order to return the top-*K* similar slides. See Methods for additional details.

In the proceeding sections, we demonstrate the performance of SISH for: (1) disease subtype retrieval using public cohorts (TCGA and CPTAC), (2) disease subtype retrieval in an independent cohort (BWH in-house data) to test generalizability, (3) anatomic site retrieval and (4) patch-level retrieval using five different datasets: colon tissue (Kather100k^45^), lung tissue (WSSS4LUAD^46^), a general morphologic atlas of digital pathology (ADP^47^), breast tissue (BCSS^48^) and prostate tissue (BWH in-house data).

## Results

### Disease subtype retrieval

**Fig. 3:**
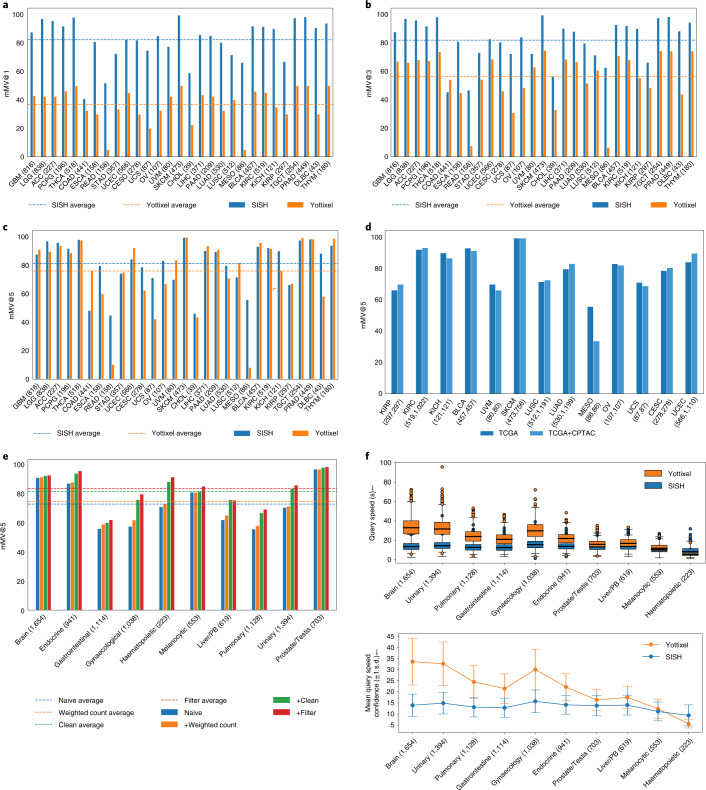
Disease subtype retrieval in public cohorts. **a**–**c**, Macro-average mMV@1,3 and 5 of SISH and Yottixel on the TCGA anatomic sites. SISH has better performance in all metrics, especially mMV@1 and mMV@3. **d**, Comparison between SISH on TCGA and TCGA+CPTAC cohorts. The performance does not vary before and after mixing with CPTAC cohorts for most cases. **e**, Ablation study result for the ranking module of SISH. We observed that SISH achieves best performance in the setting where all functions are applied (+filter) (details of each setting are described in Methods (Ablation study). **f**, Top: query speed comparison between SISH and Yottixel for each site. The box extends from the first quartile (Q1) to the third quartile (Q3) of the data and the whiskers extend from the box by 1.5× the interquartile range (IQR). Bottom: the mean confidence (±1 s.d.) of query speed between SISH and Yottixel. It is crucial to note that SISH is 2× more effective when the number of slides is over 1,000 (details on the study of speed is reported in Speed and interpretability. Numbers in parentheses denote the number of WSIs for all panels except **d**, where numbers in parentheses denote the number of WSIs in TCGA and TCGA+CPTAC, respectively. Source data

We first evaluate the performance of SISH on disease subtype retrieval using the TCGA, and we report the majority top-*k* accuracy (mMV@k, *k* = 1,3,5), which assesses how often the majority slide diagnosis in the top*-k* results matches the ground truth from the query. We use mMV@k as the primary metric for comparison because it is stricter than the commonly used top-*k* accuracy (see Methods for more details). We built the SISH pipeline on slides from each anatomic site and tested whether SISH can retrieve slides with the correct diagnosis. Overall, SISH outperformed Yottixel by achieving 45.51%, 25.62% and 5.33% higher macro-averaged mMV@1, 3 and 5, respectively, as shown in Fig. 3a–c (see detailed numerical results and individual slide retrieval results in Supplementary Tables 1 and 7). We used macro-averaging because rare cases in an unbalanced real-world histology database are as crucial as the more common ones. The improvement in performance could be partly attributed to the fact that the VQ-VAE is trained on the TCGA albeit in a self-supervised manner without using any external supervisory labels. However, the primary role of the VQ-VAE is to improve the query speed, and we investigated the role of the VQ-VAE in improving the performance (Supplementary Fig. 3). Subsequent sections demonstrate results on several datasets that were not used to train the VQ-VAE. To further investigate the results, we created the confusion matrix and Hamming distance matrix in Supplementary Fig. 1 for each site.

In addition, the speed advantage of SISH becomes pronounced especially after the number of slides in the database exceeds 1,000 (Fig. 3f). The median query speed of SISH remains near-constant despite the growing number of slides, as expected from our theoretical results analysis. We perform more experiments to demonstrate that SISH is scalable to thousands of slides (see Speed and interpretability). Since the ranking algorithm plays a crucial role in the success of SISH, we conduct an ablation study to validate all steps in the ranking module and show that SISH achieved the best performance by including all steps (red line in Fig. 3e).

We also combined kidney renal clear cell carcinoma (KIRC), uterine corpus endometrial carcinoma (UCEC), cutaneous melanoma (SKCM), lung adenocarcinoma (LUAD) and lung squamous cell carcinoma (LUSC) data from CPTAC and TCGA to test performance on a mixed public cohort with the results reported in Fig. 3d. After combining the two datasets, the distribution of the dataset over all sites became more skewed, but the performance of SISH did not vary substantially in most cases. This result further shows that SISH can address, to a degree, dataset imbalance commonly presented in the real world. The only exception was pulmonary-mesothelioma, for which the site where the disease is located was highly unbalanced. Individual retrieval results are available in Supplementary Table 8.

### Disease subtype retrieval in independent cohort

**Fig. 4:**
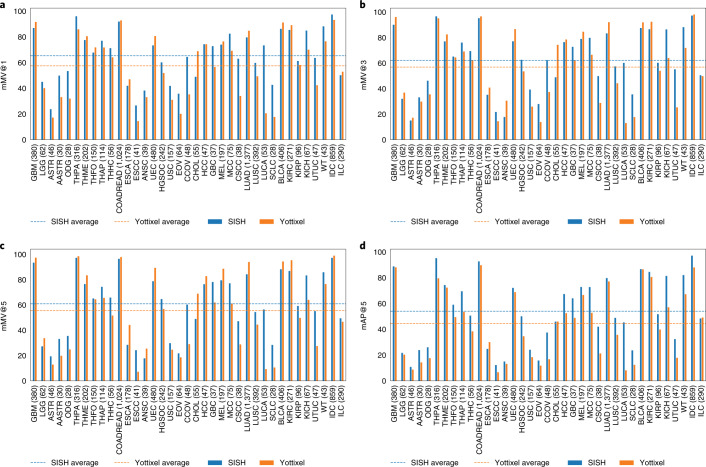
Adapting to the BWH independent test cohort. **a**–**c**, Average mMV@1, 3 and 5 scores of SISH and Yottixel for each subtype in BWH general cohorts. SISH achieved higher scores than Yottixel by 7.87%, 5.33% and 5.33% for mMV@1, 3 and 5, respectively. **d**, mAP@5 score of SISH and Yottixel. SISH outperformed Yottixel in 34 out of 37 subtypes, resulting in 9.5% higher mAP@5 score. Numbers in parentheses denote the number of WSIs. Source data

WSIs can have large scale domain shift across institutions and medical centers due to variability in tissue preparation, staining and scanning protocols. Therefore, it is essential to validate that the self-supervised VQ-VAE trained on TCGA is robust and adaptable to a different out of domain dataset. From the WSI database at Brigham and Women’s Hospital, we collected 8,035 diagnostic slides that span 9 anatomic sites with 37 primary cancer subtypes. For each anatomic site, we built our pipeline separately and reported the mMV@1,3 and 5 scores along with mean average precision at 5 (mAP@5) as a ranking-aware metric (see Evaluation in Methods). SISH performed better than Yottixel by achieving 7.87%, 5.33% and 5.33% higher macro-averaged mMV@1,3 and 5 scores, respectively, as reported in Fig. 4a–c (see numerical results and retrieval results of each slide in Supplementary Tables 2 and 9). Furthermore, SISH outperformed Yottixel in mAP@5 in 34 out of 37 subtypes, leading to a 9.5% higher macro-average mAP@5 (Fig. 4d). We also report the confusion and Hamming distance matrix for each anatomic site (Supplementary Fig. 2). Note that we did not use fine-tune or any other form of domain adaptation to refine our self-supervised encoder in this cohort, showing the generalizability of our encoder trained only on TCGA. While this adaptability mimics a common scenario where the self-supervised encoder is generically trained and the database is built for individual cohorts, we also investigated cross encoder and cross database retrieval by keeping both the self-supervised encoder and database developed on the TCGA constant and querying using cases from CPTAC and BWH data for the same set of diagnosis (see Supplementary Fig. 7). We found that while there is a drop in performance, our approach was generally adaptable.

### Rare disease subtype retrieval

**Fig. 5:**
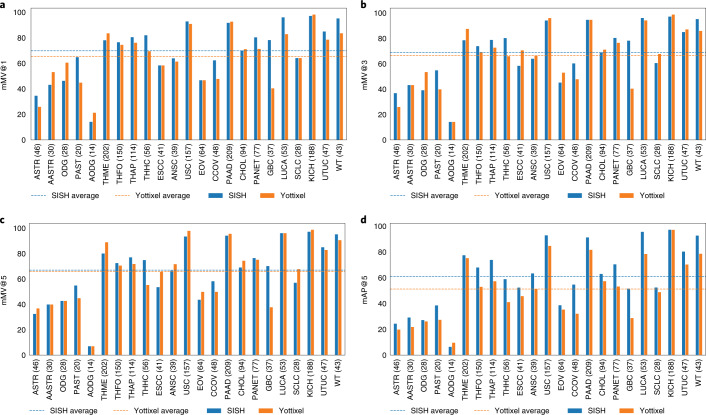
Adapting to rare cancer types. **a**–**c**, Macro-average mMV@1, 3 and 5 scores of SISH and Yottixel in each rare cancer subtype. SISH achieved higher mMV1 and 3 scores than Yottixel by 4.56% and 2.42%, respectively. **d**, Macro-average mAP@5 score. SISH outperformed Yottixel in 22 out of 23 subtypes by 9.82%. Numbers in parentheses denote the number of WSIs. Source data

The number of archival slides for rare diseases is usually fewer than that of common ones, making it challenging to train an efficient supervised classifier using modern machine learning methods. To further investigate the clinical value of SISH in assisting with the diagnosis of rare diseases, we conducted an experiment specifically on rare cancer types by combining our BWH cohort and TCGA, resulting in 1,785 slides for 23 rare-type cancers from 7 anatomic sites. The performance of SISH was on par with that of Yottixel for the mMV@5 metric, but achieved slightly better macro-averaged mMV@1 and 3 scores that were 4.56% and 2.42% higher, respectively, as shown in Fig. 5a–c (see numeric results and retrieval results of each slide in Supplementary Tables 3 and 10). Although the performance gap of mMV@1, 3 and 5 scores between SISH and Yottixel was small, SISH achieved better mAP@5 in 22 out of 23 subtypes, which resulted in an overall 9.82% performance improvement (Fig. 5d). We additionally investigate the performance of SISH against a supervised classifier trained using limited WSI data for a subset of rare diseases and found that supervised classifiers can have a high variance across cross-validation folds. SISH also performed better than the average across all folds (Supplementary Table 6).

### Anatomic site retrieval

**Fig. 6:**
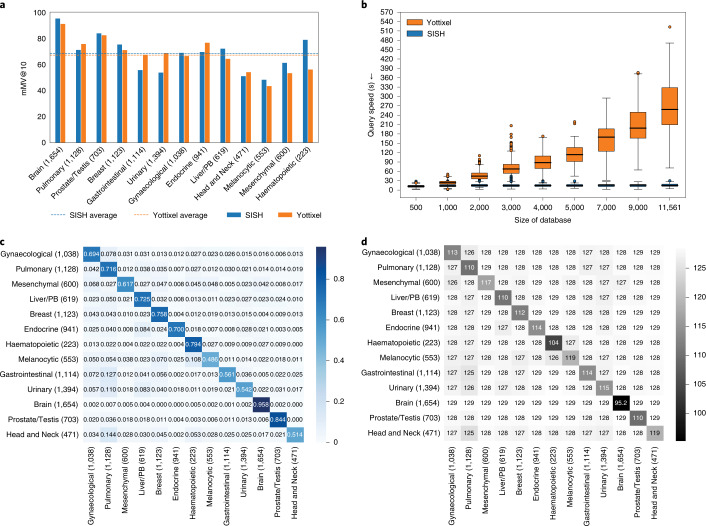
Performance on anatomic site retrieval and speed. **a**, mMV@10 comparison between SISH and Yottixel. **b**, Speed comparison between SISH and Yottixel in the TCGA anatomic site retrieval. SISH is faster than Yottixel by approximately 15× when the number of slides is over 10,000. See Speed and interpretability for more details. The box extends from Q1 to Q3 of the data and the whiskers extend from the box by 1.5 × IQR. **c**,**d**, Confusion matrix (**c**) and Hamming distance matrix (**d**) of SISH for anatomic site retrieval. The *x* and *y* axes correspond to the model prediction and ground truth, respectively. The sharp diagonal line in both matrices show that SISH can retrieve the correct results and avoid the dissimilar ones in most cases. Numbers in parentheses denote the number of WSIs from each site. PB, pancreaticobiliary. Source data

Although the anatomic site from which tissue is resected is always known, historical archival repositories that may not have corresponding digitised pathology reports and electronic medical records will significantly benefit from automated anatomic site identification. We used the diagnostic slides from TCGA and followed ref. ^36^ to group slides into 13 categories, resulting in 11,561 WSIs. We built our SISH pipeline on this database with the goal to retrieve slides with the same anatomic site as the query. SISH achieved 68.52% mMV@10 on average, which is comparable to Yottixel’s performance (67.42%) (Fig. 6a). We used mMV@10 for comparison in this experiment as this was the best performance reported in the previous study^36^. However, we note that SISH is over 15× faster than Yottixel as shown in Fig. 6b, although the classification performance gap between the two methods is small. A detailed comparison of speed between SISH and Yottixel can be found in Speed and interpretability, and individual retrieval results are available in Supplementary Table 11.

### Analysis of speed and interpretability

**Extended Data Fig. 1:**
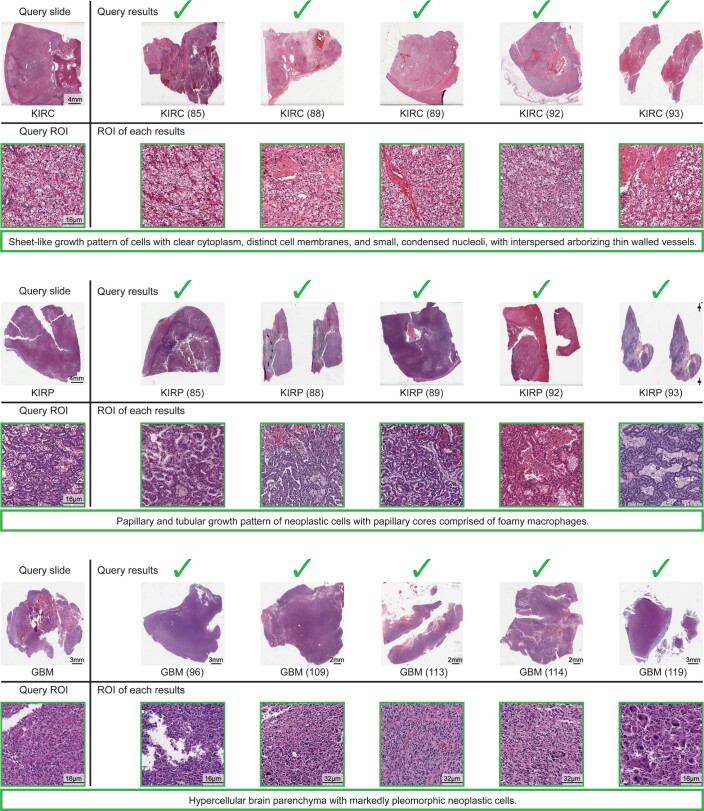
Examples of fixed-site disease subtype retrieval in TCGA cohort. Examples of retrieval slides and corresponding ROI identified by SISH in TCGA-KIRC, TCGA-KIRP, and TCGA-GBM. The green border of ROIs denotes the selected regions match the histological features annotated by the pathologist. The number in parentheses is the Hamming distance between the query slide and each result, determined by the identified ROI in each WSI. Each row shares the same scale bar unless specified otherwise.

**Extended Data Fig. 2:**
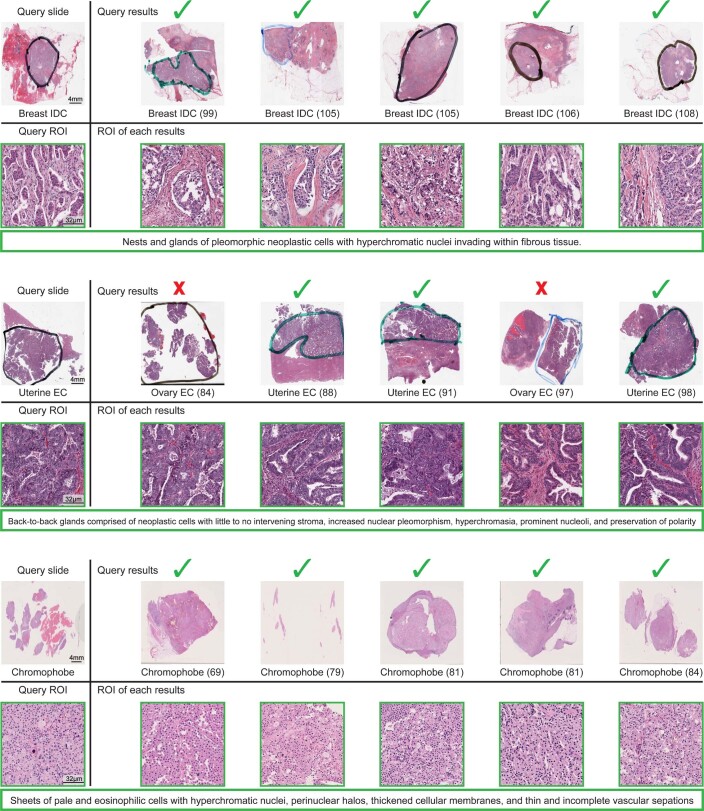
Examples of fixed-site disease subtype retrieval in independent cohort. Examples of retrieval slides and corresponding ROI identified by SISH in Breast Invasive Ductial Carcinoma (Breast IDC), Uterine Endometriod Carcinoma (Uterine EC), and Kidney Chromophobe. The green border of ROIs denotes the selected regions match the histological features annotated by the pathologist. The number in parentheses is the Hamming distance between the query slide and each result, determined by the identified ROI in each WSI. Each row shares the same scale bar unless specified otherwise. We found that SISH is sometimes confused with Ovary EC and Uterine EC, which is reasonable as both diseases have similar morphology.

**Extended Data Fig. 3:**
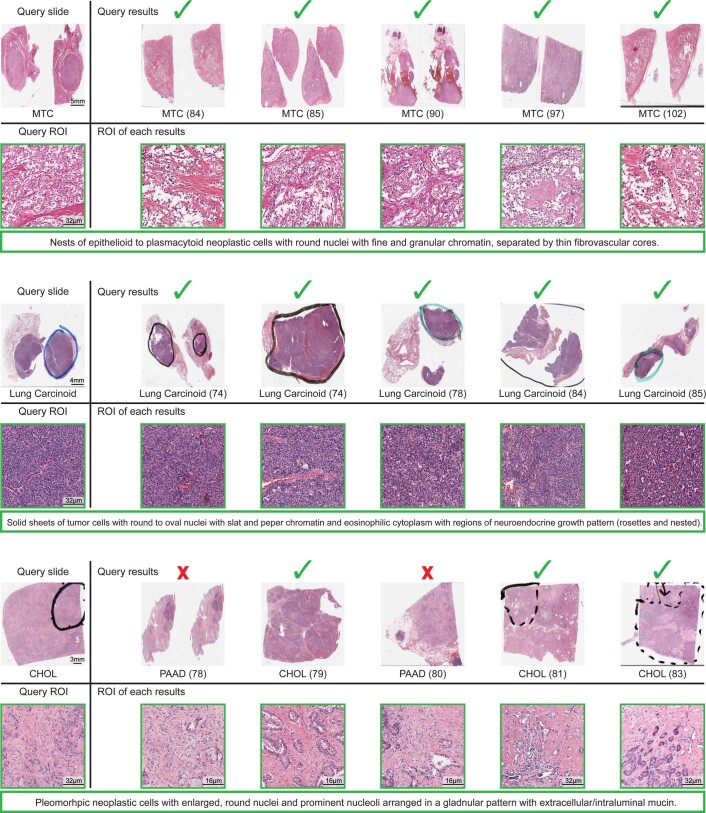
Examples of fixed-site retrieval on rare cancer subtype. Examples of retrieval slides and corresponding ROI identified by SISH in Medullary Thyroid Carcinoma (MTC), Lung Carcinoid and Cholangiocarcinoma (CHOL). The green border of ROIs denotes the selected regions match the histological features annotated by the pathologist. The number in parentheses is the Hamming distance between the query slide and each result, determined by the identified ROI in each WSI. Each row shares the same scale bar unless specified otherwise. We found that SISH is sometimes confused with Cholangiocarcinoma and Pancreatic Adenocarcinoma (PAAD), which is reasonable as both diseases have similar morphology.

**Extended Data Fig. 4:**
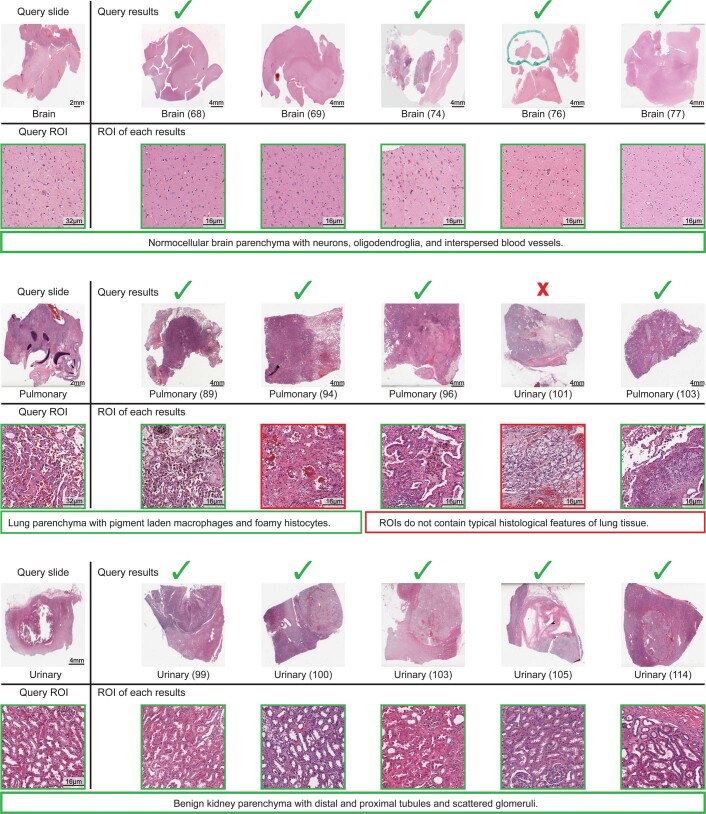
Examples of anatomic site retrieval in TCGA cohort. Examples of retrieval slides and corresponding ROI identified by SISH in Brain, Pulmonary and Kidney. The visualization showed that SISH can also identified regions that contain typical histological features for a site. The green border denotes the regions that contain typical features while the red borders denote the failure cases. The number in parentheses is the Hamming distance between the query slide and each result, determined by the identified ROI in each WSI. Each row shares the same scale bar unless specified otherwise.

We show how SISH allows the user to interpret the results of a query slide in Extended Data Fig. 1. For a query slide, SISH returns the regions in the slide that are useful for defining the similarity of the slides. This property allows us to examine these regions and ensure that the search system returns results based on reasonable morphologic evidence as agreed by pathologists instead of meaningless regions such as debris. More examples are shown in Extended Data Figs. 2–4. We conducted three interpretation studies using KIRC, ovarian serous cystadenocarcinoma (OV) and stomach adenocarcinoma (STAD) in TCGA to understand SISH’s interpretability across different levels of performance (in terms of differences in mMV@5 scores). For each study, we randomly selected 30 queries that contained at least 1 correct retrieval and then extracted the ROIs found in the query slide. We asked a board certified pathologist to rate whether the ROIs agree with their judgements by ‘agree’, ‘partially agree’ (that is, if the pathologist agrees with at least one of the ROIs) and ‘disagree’. The key finding was that the ratio of ‘agree’ plus ‘partially agree’ exceeded 70% in all studies (Supplementary Fig. 6).

We used the TCGA dataset from the anatomic site retrieval experiment to evaluate query speed. We applied weighted sampling to select slides from each site to create databases of size 500, 1,000, 2,000, 3,000, 4,000, 5,000, 7,000 and 9,000, and the overall dataset with 11,561 slides. We implemented both methods in Python and evaluated them on the same machine for fair comparison. The average query speeds of both methods are reported in Fig. 6b. Since we observed that Yottixel was inefficient beyond 3,000 slides, we used the same 100 queries sampled from the databases to calculate the average query speed of SISH and Yottixel instead of using all data when the size of the database exceeded 3,000. By comparison, the average query speed of SISH remained almost constant, with low variances across the range of database sizes, consistent with our theoretical results. This result demonstrates that SISH can scale with the growing number of slides while maintaining a relatively constant query speed.

### Patch-level retrieval

For patch-level retrieval, we viewed each patch query as a single mosaic fed into the SISH search pipeline. Since there is only one patch in the mosaic, there is no need for the ranking module. We obtained the top-*K* results by directly sorting the predictions by their Hamming distances. We collected patch data across 4 anatomic sites (lung, breast, colon and prostate) from 6 cohorts: TCGA, BWH, Guangdong Provincial People’s Hospital (GDPH), Atlas, National Center for Tumor Diseases (NCT) biobank, and University Medical Center Mannheim (UMM). We evaluated SISH on 10 tissue types and built database sizes from 4,000 to 13.4 million from the collected patch data. More details can be found in the Methods. We only compared SISH to Yottixel as neither was specifically designed for patch retrieval, unlike other published methods.

**Fig. 7:**
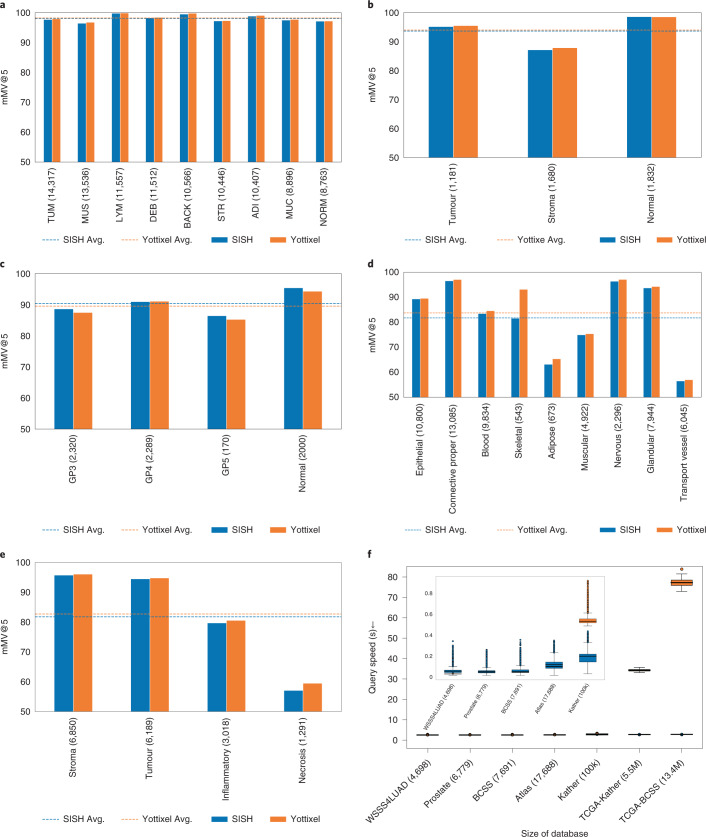
Patch-level retrieval. **a**–**e**, mMV@5 results for SISH and Yottixel for patch level retrieval on multiple patch level datasets: Kather100k, WSSS4LUAD, BWH prostate, Atlas and BCSS datasets. SISH performed similarly to Yottixel on all datasets. **f**, Query speeds of SISH and Yottixel. SISH achieved faster mean query speed by a factor of 3 to 230 as the size of the database grew from 100,000 to 13.4 million images. The results were averaged across query times of all data in the database, except for the TCGA-Kather and TCGA-BCSS databases because of their large size. For these latter two databases, the results were averaged across the query time of all data in Kather100k and BCSS for SISH and 100 random samples from Kather100k and BCSS for Yottixel due to slow performance. In summary, SISH has similar performance to Yottixel but faster search speed when the size of the database grows. Inset: a closer look at the first five patch dataset. The box extends from Q1 to Q3 of the data and the whiskers extend from the box by 1.5 × IQR. Source data

**Extended Data Fig. 5:**
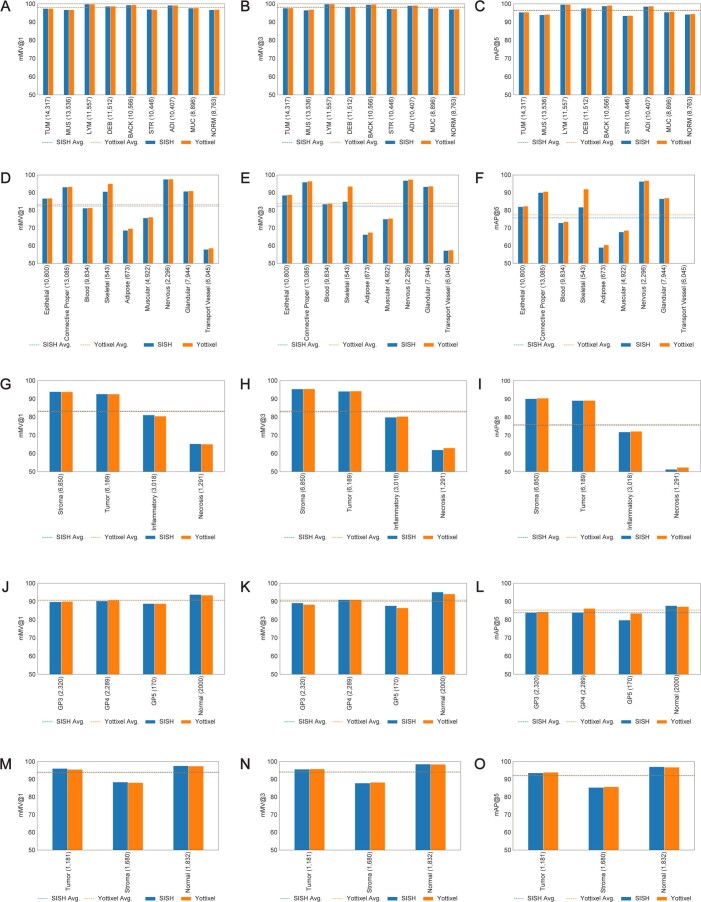
mMV@1, mMV@3 and mAP@5 results on patch data. A-C: Kather100k, D-F Atlas, G-I Breast, J-L BWH prostate and M-O WSSS4LUAD. On all datasets and metrics, the performance of SISH was on par with Yottixel.

**Extended Data Fig. 6:**
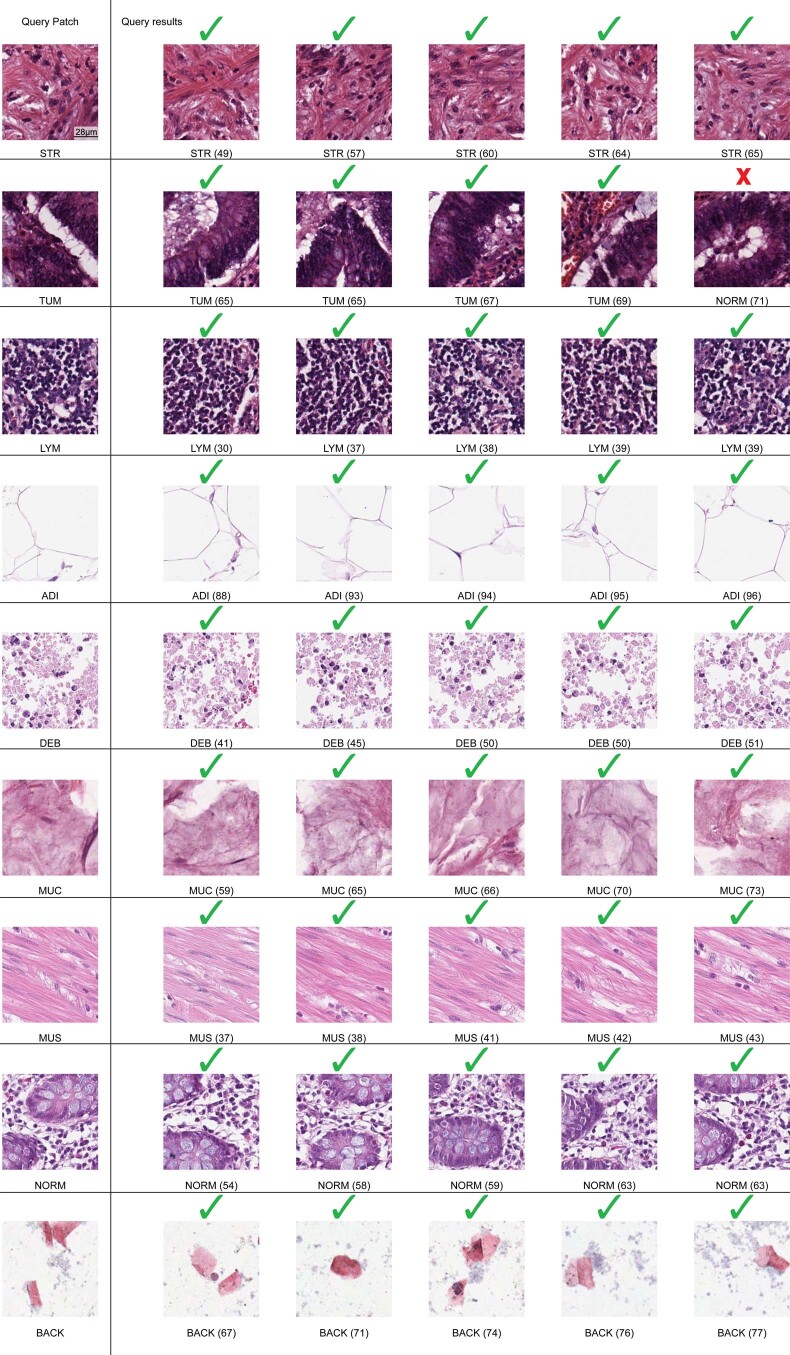
Examples of patch retrieval on Kather100k colon data. The patches of cancer associated stroma (STR), colorectal adenocarcinoma epithelium (TUM), lymphocytes (LYM), adipose (ADI), debris (DEB), mucus (MUC), muscle (MUS), normal tissue (NORM), and background (BACK) are presented in the figure. The number in parentheses is the Hamming distance between the query patch and each result. All patches share the same scale bar.

**Extended Data Fig. 7:**
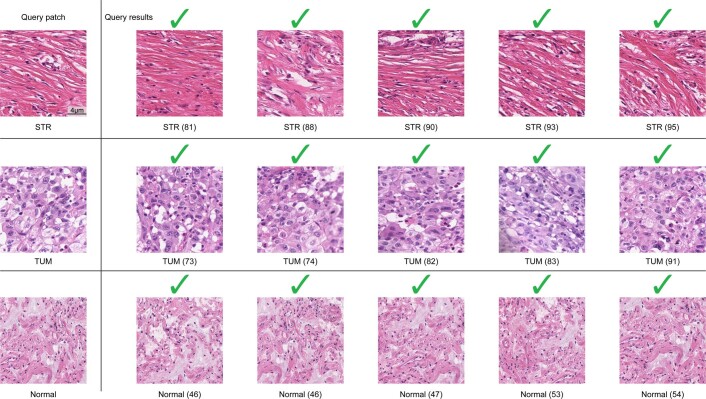
Examples of patch retrieval on WSSS4LUAD lung data. We considered three types of tissues in WSSS4LUAD data (i.e., Stroma (STR), Tumor (TUM) and Normal (non-neoplastic)). The number in parentheses is the Hamming distance between the query patch and each result. All patches share the same scale bar.

**Extended Data Fig. 8:**
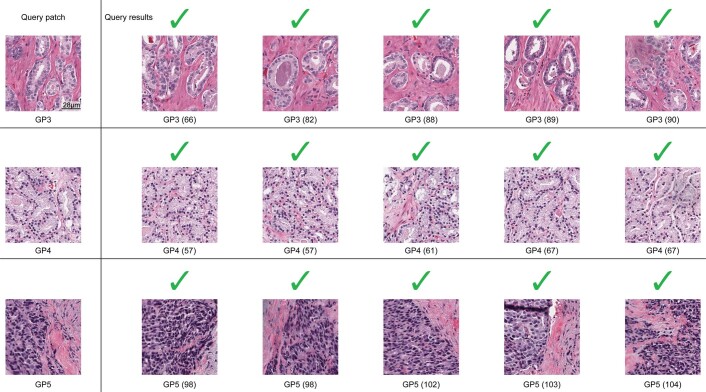
Examples of patch retrieval on in-house BWH prostate data. The patches of Gleason pattern (GP) 3, 4 and 5 are presented in the figure. The number in parentheses is the Hamming distance between the query patch and each result. All patches share the same scale bar.

**Extended Data Fig. 9:**
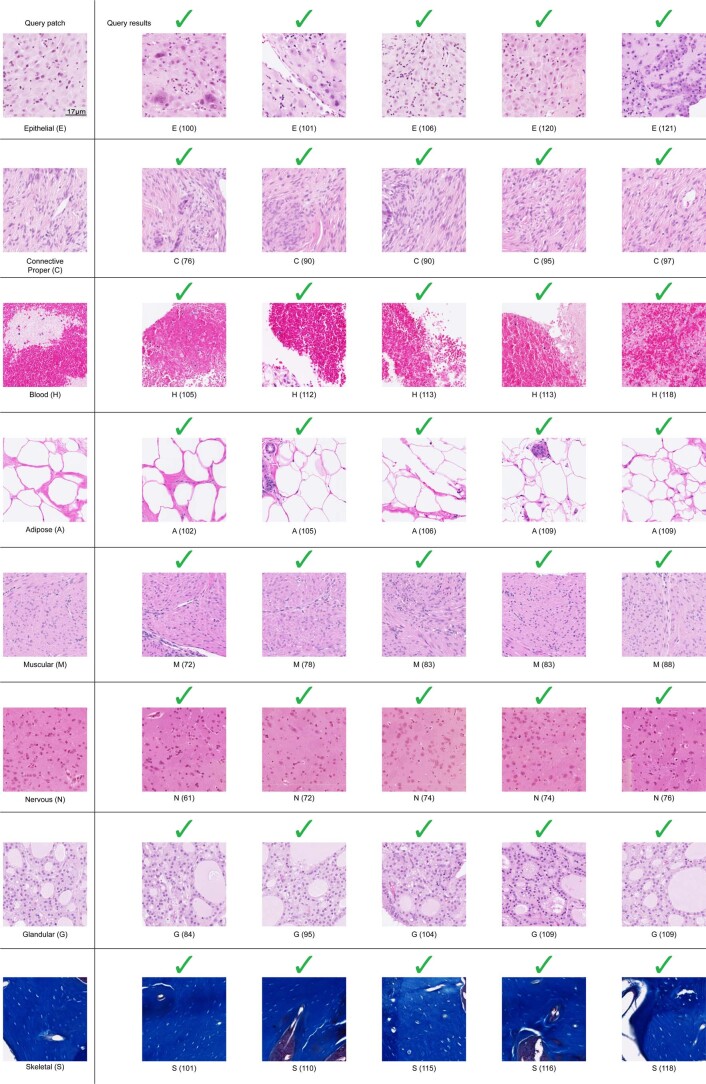
Examples of patch retrieval on Atlas data. The patches of Epithelial (E), Connective Proper (C), Blood (H), Adipose (A), Muscular (M), Nervous (N), Glandular (G) and Skeletal (S) are presented in the figure. The tissue patches are collected from various unknown organs^47^. The number in parentheses is the Hamming distance between the query patch and each result. All patches share the same scale bar.

**Extended Data Fig. 10:**
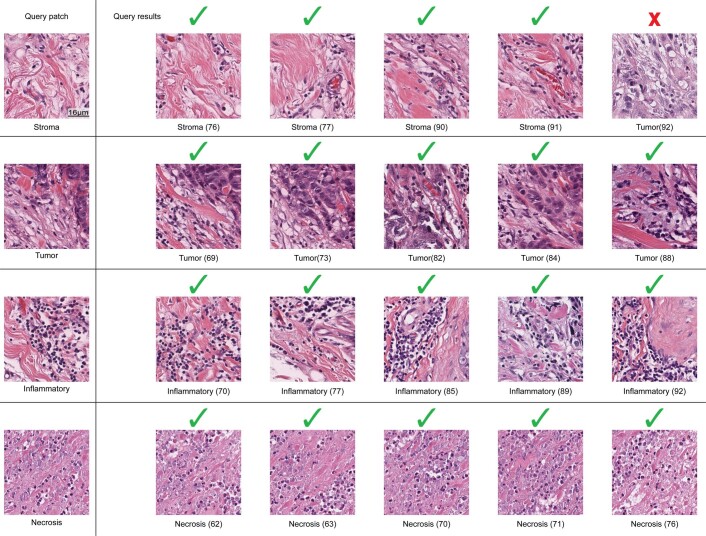
Examples of patch retrieval on BCSS breast data. The patches of Stroma (STR), Tumor (TUM), Inflammatory, and Necrosis are presented in the figure. The number in parentheses is the Hamming distance between the query patch and each result. All patches share the same scale bar.

We report the mMV@5 patch retrieval performance of Kather100k^45^, WSSS4LUAD^46^, BWH prostate, Atlas^47^ and BCSS^48^ in Fig. 7a–e. The performance of SISH is on par with that of Yottixel in all experiments, SISH’s mean query speed was faster than Yottixel’s by three times when the size of the database reached 100,000, as shown in Fig. 7f (0.18 ± 0.06 s versus 0.53 ± 0.04 s). To further test the query speed on larger databases, we created two large databases by mixing Kather100k with colon adenocarcinoma and rectal adenocarcinoma in TCGA (TCGA-Kather) and BCSS with Breast in TCGA (TCGA-BCSS). Both TCGA cohorts were patched at 256 × 256. The size of the merged databases reached 5.5 million and 13.4 million images, respectively. The mean query speed of SISH reached 120 times faster than Yottixel on TCGA-Kather and 230 times faster on TCGA-BCSS (0.27 ± 0.036 s versus 32.44 ± 1.02 s and 0.32 ± 0.016 s versus 74.55 ± 2.00 s), as shown in Fig. 7f. On databases of size over 10 million, we observed a performance improvement in speed over previous approaches^34^. We report more comparisons with different metrics and show examples in Extended Data Figs. 5–10 (see individual retrieval results in Supplementary Tables 12–16).

## Discussion

In summary, we show that SISH addresses several of the key challenges in whole-slide image search: speed, accuracy and scalability. Our experiments demonstrate that SISH is an interpretable histology image search pipeline that achieves constant search speed after training with only slide-level labels. We also demonstrate that SISH has strong performance on large and diverse datasets, can generalize to independent cohorts as well as rare diseases and, finally, that it can be used as a search engine not just for WSIs, but also for image patch retrieval.

Search functions have transformed and enabled our modern digital lives, and digitization of pathology reports (as well as medical records themselves) represent a similar transformation for pathology practices. Searching databases of digitized pathology report texts has proved to be extremely useful in clinical practice, from seeing how colleagues have signed out similar cases to identifying cases for research or quality control initiatives. However, pathology reports capture only a tiny portion of the information contained in the associated slides, and that information can be quite limited. Depending on the pathology practice, there may be little description of the tissue on a set of slides beyond only what was necessary to make a particular diagnosis or was required for structured/synoptic reporting. Additionally, what descriptions are present using the language of pathology (for example, ‘myxoid’, ‘salt-and-pepper chromatin’ or ‘micropapillary’) are not localized to a particular slide or region of interest.

By comparison, a search function that harnesses the rich, spatially resolved information in pathology images is much more powerful for certain applications. Use-cases of such a system include: (1) pathology trainees finding cases with similar morphologies to learn how their mentors would diagnose the case of interest; (2) disease subtyping to provide more evidence to a pathologist for a particular diagnosis; (3) researchers identifying tumours that share certain features for clinical or genomic correlations; (4) assistance in diagnosis of rare morphological findings; (5) quality control functions to identify potential sample swaps, patient misidentification, or outlier detection; and (6) primary site suggestions for metastases (7) disentangling large historical repositories in the absence of electronic medical and pathology reports.

Additionally, the importance of performant image search systems will probably increase in the near future, as the penetrance of slide scanning solutions in pathology practices grows. As institutions’ WSI repositories grow to hundreds of thousands or millions of slides, only systems with constant or near-constant search speed and the ability to operate without pixel-level annotations will reasonably be able to scale and will be deployed for use in clinical practice. Interpretability of such a system will allow quality control of the search itself to be baked in, giving users the ability to troubleshoot searches on the fly. A caveat, however, to the interpretability of SISH is that it only returns relevant ROIs and as with many deep learning systems the feature representation itself largely remains abstract^49^.

Our approach has several other limitations that future studies may address. The discrete index used to look up similar candidate patches may have limited expressive power, especially if a query mosaic is large, as this may require visiting many neighbors in the vEB tree to cover all promising candidates. This can limit the efficiency of the search engine despite a theoretical constant search time. Additionally, by using a fixed set of patches to represent each WSI in the form of a mosaic, we could potentially miss other informative regions in the slide that are valuable for search but do not make it into the final mosaic of the WSI. Also, during retrieval, candidates for each patch in the query mosaic are mutually exclusive of one another; since all patches are extracted at a single fixed resolution, morphological patterns that span larger regions of interest may not be adequately represented during search. Other limitations include the large space (memory) complexity of the vEB tree; however, we found the memory utility to be reasonable and tractable on a consumer grade workstation for all experiments conducted in this study. Given the scale of the study, several hyper-parameters were not tuned and conventional values were used from prior literature; further tuning these hyper-parameters may lead to better results.

Finally, the current system has been developed only to search for images using a query image. In clinical practice, pathologists rely on other data such as the patient’s medical record, other imaging modalities and molecular test results to guide diagnoses and clinical decision making. Therefore, we believe one important future direction is to develop a multimodal version of SISH by pairing each WSI with other data from the same patient so that our search system can present a holistic view for pathologists, given a query WSI. In a similar vein, extending SISH to accept multimodal queries, such as text or genomic data, would be a promising direction, provided an efficient way to compare and find semantic similarities between disparate and orthogonal data types can be developed. Other advances could include the development of similar fast and scalable search engines for multiplex immunofluorescence and spatial transcriptomics data. Overall, SISH represents an initial proof-of-concept for the utility of self-supervised learning for retrieval in massively large medical datasets and paves the way for future studies to explore the utility of larger datasets at scale, and additional modalities to eventually build a universal search engine for biomedicine.

## Methods

### SISH

SISH is a histology-image search pipeline that addresses the scalability issues of speed, storage and pixel-wise label scarcity. While image-level class labels or annotation for whether a pair or triplet of images are similar/dissimilar has often been leveraged to explicitly learn an embedding space that captures semantic similarity between images, or to identify key points before retrieval^50,51^, it is difficult to directly apply such techniques to WSI image search due to the high dimensionality of large WSIs and the lack of patch-level annotation. Instead, SISH builds upon a set of preprocessed mosaics from WSIs without pixel-wise or ROI-level labels to reduce storage and labelling costs, by relying on indices learned via self-supervised learning and pretrained embeddings. SISH scales with a constant search speed, regardless of the size of the database, by taking advantage of the benefits of discrete latent codes from a VQ-VAE, and using guided search and ranking algorithms. We present these essential components of SISH in this section and provide a pictorial illustration for the methods described in Fig. 2. For clarity, we have summarized all symbols used in the following text in Supplementary Table 18.

#### Discrete latent code of VQ-VAE

VQ-VAE^41^ is a variant of a Variational AutoEncoder (VAE) that introduces a training objective that allows for discrete latent codes. Let ${{{\boldsymbol{e}}}}\in {{\mathbb{R}}}^{K\times D}$ be the latent space (that is, codebook) where *K* is the number of discrete codewords and *D* is the dimension of the codewords. We set *K* = 128 and *D* = 256 in our experiments. To decide the codeword of the given input, an encoder *q* encodes input *x* as *z*_*e*_(*x*). The final codeword *z*_*q*_(*x*) of *x* and the training objective function are given by

1 $${{{{\boldsymbol{z}}}}}_{q}(x)={{{{\boldsymbol{e}}}}}_{k},{{{\rm{where}}}}\ k={{{{\rm{argmin}}}}}_{j}\left\Vert {{{\boldsymbol{{z}}}_{e}}}({{{\boldsymbol{x}}}})-{{{{\boldsymbol{e}}}}}_{j}\right\Vert ,$$

2 $$\log p({{{\boldsymbol{x}}}}| {{{{\boldsymbol{z}}}}}_{q}({{{\boldsymbol{x}}}}))+\left\Vert {{{\rm{sg}}}}[{{{\boldsymbol{{z}}}_{e}}}({{{\boldsymbol{x}}}})]-{{{\boldsymbol{e}}}}\right\Vert +\alpha \left\Vert {{{\boldsymbol{{z}}}_{e}}}({{{\boldsymbol{x}}}})-{{{\rm{sg}}}}[{{{\boldsymbol{e}}}}]\right\Vert ,$$

where *α* is a hyperparameter and sg denotes the stop gradient operation. A stop gradient operation acts as the identity function during the forward pass while having zero gradient during the backward pass. The first term in the objective function optimizes the reconstruction of the encoder and decoder, the second term is used to update the codebook, and the third term is used to prevent the encoder’s output from diverging too far from the latent space. The architecture of our VQ-VAE model is shown in detail in Supplementary Fig. 4. We re-ordered the codebook on the basis of the value of the first principal component and changed the latent code accordingly as we found that the re-ordered codebook can provide a more semantic representation of the original input image (see Supplementary Fig. 5).

#### Feature extraction, index generation and index encoding

We show how each patch *i* in the mosaic of a WSI can be represented by a tuple (*p*_*i*_, *h*_*i*_) composed of a patch index *p*_*i*_ and a patch texture feature *h*_*i*_. To get *p*_*i*_, we encode and re-map the latent code *z*_*i*_ from the encoder and re-ordered codebook from the VQ-VAE. The index *p*_*i*_ is determined by the following equations:

3 $${{{{\boldsymbol{z}}}}}_{i,1}={{{\rm{AVGPOOL}}}}(2,2)({{{{\boldsymbol{z}}}}}_{i})$$

4 $${{{{\boldsymbol{z}}}}}_{i,2}={{{\rm{AVGPOOL}}}}(2,2)({{{{\boldsymbol{z}}}}}_{i,1})$$

5 $${{{{\boldsymbol{z}}}}}_{i,3}={{{\rm{AVGPOOL}}}}(2,2)({{{{\boldsymbol{z}}}}}_{i,2})$$

6 $${p}_{i}={{{\rm{SUM}}}}({{{{\boldsymbol{z}}}}}_{i,1})+{{{\rm{SUM}}}}({{{{\boldsymbol{z}}}}}_{i,2})\times 1{0}^{6}+{{{\rm{SUM}}}}({{{{\boldsymbol{z}}}}}_{i,3})\times 1{0}^{11}$$

7 $${{{\rm{SUM}}}}({{{{\boldsymbol{z}}}}}_{{{{\boldsymbol{i,1}}}}})\in [0,130048],{{{\rm{SUM}}}}({{{{\boldsymbol{z}}}}}_{{{{\boldsymbol{i,2}}}}})\in [0,32512],{{{\rm{SUM}}}}({{{{\boldsymbol{z}}}}}_{{{{\boldsymbol{i,3}}}}})\in [0,8128]$$

To convert the information in the latent code from higher to lower resolution, we apply a series of 2 × 2 average pooling. We then take the summation to aggregate the latent code in each resolution, as the summation operator has better expressive power than the mean or maximum^52^. We get the final integer index by taking the summation of the information aggregated in each resolution and multiplying it by 10^0^, 10^6^ and 10^11^, respectively. The intuition behind choosing the power is to keep the information of the latent code in each resolution (that is, *z*_*i*,1_, *z*_*i*,2_ and *z*_*i*,3_) separate. For example, multiplying Sum(*z*_*i*,2_) by 10^6^ separates the feature in the second layer from Sum(*z*_*i*,1_) since the maximum of the latter is 130,048. Likewise, multiplying Sum(*z*_*i*,3_) by 10^11^ separates the feature in the last layer from the previous two. We insert each *p*_*i*_ into the vEB tree for fast search. We apply this process to WSIs in the datasets to build our databases. Note that the time complexity of all operations in the vEB tree is $O(\log \log (M))$. On the basis of the properties of the vEB tree, *M* can be determined by

8 $$M={2}^{x} > \max ({p}_{i})\,,$$

where *x* is the minimum integer that satisfies the inequality. Since our codebook size ranges from 0 to 127, we can determine the maximum summation Sum(*z*) in each level. Solving the inequality, we find that the minimum *M* that satisfies the inequality is *M* = 1,125,899,906,842,624. Because *M* is a constant that only depends on the index generation pipeline, our search performance is *O*(1). One limitation of using vEB is that it has a large space complexity *O*(*M*) where *M* depends on the size of the codebook and the dynamic range of the index used for search. *M* remains fixed and does not scale with the number of data points (WSIs or patches) in the database. To get *h*_*i*_, we use DenseNet121 to extract features from the 1,024 × 1,024 patch at ×20, then follow the algorithm proposed in ref. ^36^ to binarize it (that is, starting from *∞*; if the next value is smaller than the current one, the current value 0 is assigned, and 1 is assigned otherwise).

In addition to creating the tuple to represent the patch, we also make a hash table *H* with *p*_*i*_ as key and the metadata *μ* of the patch as value. The metadata include the texture feature *h*_*i*_, the name of the slide associated with the patch, the coordinates on the slide from which the patch is cropped, the file format of the slide and the diagnosis of the slide. Note that different patches could share the same key. In this case, the value is a list that stores the metadata for each patch. If the size of the search database is significantly large, which is expected to be the case for most practical real-world databases, the search speed would be greater than pre- and post-processing steps. When running a fixed number of iterations, the *K*-means clustering algorithm (Lloyd’s algorithm) has time complexity *O*(*BKIC*) where *B* is the number of patches in a WSI, *K* is the number of cluster centroids, *I* is the number of iterations and *C* is the dimension of each input data point. For fixed *I*, *K* and *C*, the initial clustering steps of mosaic construction is O(*B*). To obtain the final mosaic, a fixed percentage (e.g. 5%) of patches are sampled from each cluster, and hence the resulting mosaic varies from slide to slide with size *B*′ = 0.05 × *B*. During retrieval, the number of total candidates proposed (before ranking) is *T* · (*k*_succ_ + *k*_pred_) · *B*′ (see the next section for the definition of *T*, *k*_succ_ and *k*_pred_). For ranking, the complexity is *O*(*B*′). Therefore, given fixed *k*_succ_, *k*_pred_ and *T*, the time complexity of retrieval is *O*(*B*′). Note that since the size of a WSI is capped by the physical size of the glass slide and the tissue specimen, for a fixed patch size, we can always pick a reasonable constant *B*_max_ to upper bound the maximum *B* in the database and in incoming query slides. Therefore, the entire workflow has a theoretical constant time complexity of *O*(1). In real-world scenarios where we expect the size of the database to scale to hundreds of thousands or millions of slides, the time complexity of retrieval will dominate over other steps such as mosaic generation and ranking if we do not use an *O*(1) search algorithm and it instead scales with *O*(*n*) or *O*(*n*log*n*), where *n* is the size of the database. However, we note that while in most practical scenarios with increasingly large databases, the size of the WSI database (*n*) would be larger than the size of the number of patches in the query slide (*B*); in rare cases where the size of the database is very small, such that average *B* is not negligible compared to *n*, while the search operation will continue to have a constant *O*(1) complexity, the speed of the overall pipeline may be limited by the mosaic generation *O*(*B*_max_). Mosaic generation can also be completed before case review, further improving search speeds.

#### Guided search algorithm

For clarity, we use *m*_*i*_ to denote the patch index in the mosaic of the query slide to distinguish those in the database. Given a query slide *I* represented as *I* = {(*m*_1_, *h*_1_), (*m*_2_, *h*_2_), …, (*m*_*k*_, *h*_*k*_)} with *k* patches, where each tuple is composed of the index of the patch *m*_*i*_ and its texture features *h*_*i*_, we apply guided-search to each tuple and return the corresponding results *r*_*i*_. The output takes the form of *R*_*I*_ = {*r*_1_, *r*_2_, …, *r*_*k*_}. Each *r*_*i*_ = {(*p*_*i*1_, *μ*_*i*1_), (*p*_*i*2_, *μ*_*i*2_), …, (*p*_*in*_, *μ*_*in*_)}, a set of tuples consisting of the indices of similar patches (*p*_*i*1_, *p*_*i*2_, …, *p*_*in*_) and their associated metadata (*μ*_*i*1_, *μ*_*i*2_, …, *μ*_*in*_). *μ*_*ij*_ includes all metadata associated with the *j*-th patch plus the Hamming distance between *h*_*i*_ and *h*_*j*_. A visual illustration is shown in Fig. 2.

The drawback to using only *m*_*i*_ for the query is that the current patch index is sensitive to minor changes in *z*_*i*,3_. For example, a patch that differs from another by 1 incurs a 10^11^ difference in index, putting the two patches far from each other in the vEB tree. To address this issue, we create a set of candidate indices *m*_*i*,*c*+_ and *m*_*i*,*c*−_ along with the original *m*_*i*_ by adding and subtracting an integer *C* for *T* times from Sum(*z*_*i*,3_). We call helper functions forward-search and backward-search to search the neighbour indices in *m*_*i*,*c*+_ and *m*_*i*,*c*−_, respectively. Both functions include only those neighbouring indices whose Hamming distance from the query *h*_*i*_ is smaller than a threshold, *θ*_*h*_. The details of these algorithms are presented in Algorithms 1 through 3.

**Algorithm 1 Guided Search Algorithm**

 *H* ← *hash table*     ⊳ Hash table with patch index as key and metadata as value

 *C*, *T* ← 50 × 10^11^, 10      ⊳ Integer and number of times for addition and subtraction

 *θ*_*h*_ ← 128    ⊳ Threshold of the Hamming distance between query patch index and the neighbor

 *k*_succ_, *k*_pred_ ← 375     ⊳ Number of time to call *vEB.Successor() and vEB.Predecessor()*

 **Function** GUIDED-SEARCH(*m*_*i*_, *h*_*i*_, *C*, *T*, *θ*_*h*_, *k*_pred_, *k*_succ_, *H*, *vEB*)

 *m*_*i*,*c*+_, *m*_*i*,*c*−_, *results* ← {}, {}, {}

 *V* ← {}

 *m*_*i*,*c*+_. *insert*(*m*_*i*_)

 **for** *t* ← 1, 2, . . . , *T* **do**

  *m*_tmp+_, *m*_tmp-_ ← *m*_*i*_ + *t* × *C*, *m*_*i*_ − *t* × *C*

  *m*_*i*,*c*+_. *insert*(*m*_tmp+_)

  *m*_*i*,*c*−_. *insert*(*m*_tmp-_)

 *results*_+_, *V* ← FORWARD-SEARCH(*m*_*i*,*c*+_, *k*_succ_, *θ*_*h*_, *V*, *H*, *vEB*)

 *results*_−_ ← BACKWARD-SEARCH(*m*_*i*,*c*−_, *k*_pred_, *θ*_*h*_, *V*, *H*, *vEB*)

 *results*. *insert*(*results*_+_)

 *results*. *insert*(*results*_−_)

 *results* ← SORT-ASCENDING(*results*, *key* = *results*. *hammingdistance*)

 **return** *results*

#### Results ranking algorithm

Our ranking function Ranking (Algorithm 4) takes the results *R*_*I*_ = {*r*_1_, *r*_2_, …, *r*_*k*_} from Guided-Search as input. The output is the top-*k* similar slides given the query slide *I*. We set *k* equal to 5 for all experiments, except for anatomic site retrieval where *k* is equal to 10. The intuition behind Ranking is to find the most promising patches in *R*_*I*_ on the basis of the uncertainty. It relies on three helper functions—Weighted-Uncertainty-Cal (Algorithm 5), Clean (Algorithm 6) and Filtered-By-Prediction (Algorithm 7).

Weighted-Uncertainty-Cal (Algorithm 5) takes *R*_*I*_ as input and calculates the uncertainty for each *r*_*i*_ by computing their entropy (that is, frequency of occurrence of slide labels). The lower the entropy, the less uncertain the patch and vice versa. The output is the entropy of *r*_*i*_, along with records that summarize the diagnosis occurrences and Hamming distance of each element in *r*_*i*_. The disadvantage of counting the occurrences naively in the entropy calculation is that the most frequent diagnosis in the anatomic site dominates the result and therefore downplays the importance of others. We introduce a weighted occurrence approach to address this issue. The approach counts the diagnosis occurrences by considering the percentage of the diagnosis in the given site and the diagnosis’s position in the retrieval results. It calculates the weight of each diagnosis in the anatomic sites by the reciprocal of the number of diagnosis. We normalize the weights such that their summation is equal to a constant *N*. A diagnosis’s final value in *r*_*i*_ is the normalized weight of the diagnosis multiplied by the inverse of position where the diagnosis appears in *r*_*i*_. Therefore, the same diagnosis can have different weighted occurrences because of its position in *r*_*i*_. As such, less frequent diagnoses and those with lower Hamming distance (that is, close to the beginning of the retrieval results) gain more importance in the ranking process. As illustrated in Fig. 2, we summarize *R*_*I*_ with three metadata values, *S*_*lb*_, *S*_*m*_ and *S*_*l*_, to facilitate the subsequent processes. Specifically, *S*_*m*_ is a list that stores tuples of form (index of *r*_*i*_, entropy, Hamming distance info in *μ*_*ij*_, length of *r*_*i*_), *S*_*l*_ is an array that only stores the length of *r*_*i*_ and *S*_*lb*_ is a nested dictionary that stores the disease occurrences in *r*_*i*_.

**Algorithm 2 Forward Search Algorithm**

 **function** Forward-Search(*m*_*i*,*c*+_, *k*_succ_, *θ*_*h*_, *V*, *H*, *vEB*)

 *res*_+_ ← {}

 **for** *i*_+_ in *m*_*i*,*c*+_ **do**

  *succ*_*cnt*, *succ*_prev_ ← 0, *i*_+_

  **while** *succ*_*cnt* < *k*_*succ*_**do**

   *succ* ← *vEB*. *Successor*(*succ*_prev_)

   **if** *succ* ∈ *V* or *succ* is empty **then**

    break

   **else if** *H*[*succ*]. *len*() = = 0 **then**

    // The case when the patient is identical to query slide *I*

    *succ*_prev_ ← *succ*

    continue

   **else**

    // Find the patch with smallest Hamming distance in the same key

    *dist*_*j*_, *j* ← Argmin_*j*_(Hamming-Distance(*h*_*i*_, *H*[*succ*]))

   **if** *dist*_*j*_ < *θ*_*h*_ **then**

    *V*. *insert*(*succ*)

    *meta* ← *H*[*succ*][*j*]

    *res*_+_. *insert*((*dist*_*j*_, *meta*))

   *succ*_*cnt*, *succ*_prev_ ← *succ*_*cnt* + 1, *succ*

 **return** *res*_+_, *V*

**Algorithm 3 Backward Search Algorithm**

 **function** Backward-Search(*m*_*i*,*c*−_, *k*_pred_, *θ*_*h*_, *V*, *H*, *vEB*)

 *res*_−_ ← {}

 **for** *i*_−_ in *m*_*i*,*c*−_**do**

  *pred*_*cnt*, *pred*_prev_ ← 0, *i*_−_

  **while** *pred*_*cnt* < *k*_pred_ **do**

   *pred* ← *vEB*. *Predecessor*(*pred*_prev_)

   **if** *pred* ∈ *V* or *pred* is empty **then**

    break

   **else if** *H*[*pred*]. *len*() = = 0 **then**

    // The case when the patient is identical to query slide *I*

    *pred*_prev_ ← *pred*

    continue

   **else**

    // Find the mosaic with smallest Hamming distance in the same key

    *dist*_*j*_, *j* ← Argmin_*j*_(Hamming-Distance(*h*_*i*_, *H*[*pred*]))

   **if** *dist*_*j*_ < *θ*_*h*_ **then**

    *V*. *insert*(*pred*)

    *meta* ← *H*[*pred*][*j*]

    *res*_−_. *insert*((*dist*_*j*_, *meta*))

   *pred*_*cnt*, *pred*_prev_ ← *pred*_*cnt* + 1, *pred*

 **return** *res*_−_

**Algorithm 4 Results Ranking Algorithm**

 **function** RANKING(*R*_*s*_, *D*_*inv*, *N*, *K*)

 **if** *R*_*s*_ is empty **then return**

 *D*_*inv* ← NORMALIZE(*D*_*inv*, *N*) ⊳ Normalize the reciprocal of diagnosis count so that the sum is equal to N. *N* = 10 for the fixed site and *N* = 30 for the anatomic site experiments, respectively.

 *S*_*lb*_, *S*_*m*_ ← {}, {}

 *S*_*l*_ ← {}

 **for** each patch’s results *r*_*i*_ in *R*_*S*_ **do**

  **if** *r*_*i*_ is not empty **then**

   *Ent*, *label*_*cnt*, *dist* ← WEIGHTED-UNCERTAINTY-CAL(*r*_*i*_, *D*_*inv*)

   *S*_*lb*_. *insert*(*i*, *label*_*cnt*)

   *S*_*m*_. *insert*((*i*, *Ent*, *dist*, *r*_*i*_. *len*()))

   *S*_*l*_. *insert*(*r*_*i*_. *len*())

  **else**

   continue

 *S*_*m*_ ← CLEAN(*S*_*m*_, *S*_*l*_)

 *f* ← FILTERED-BY-PREDICTION(*S*_*m*_, *S*_*lb*_)

 *R*_*ret*_, *V* ← {}, {}

 **for** *e* in *S*_*m*_ **do**

  *uncertainty*, *i* ← *e*. *Ent*, *e*. *i*

  **if** *i* in *f* **then**

  continue

  **else**

   *r*_*i*_ = *R*_*S*_[*i*]

   **for** *p*, *μ* in *r*_*i*_ **do**

    **if** *uncertainty* == 0 and *μ*. *slide*_*name* not in *V* **then**

     *R*_*ret*_. *insert*(*μ*)

     *V*. *insert*(*μ*. *slide*_*name*)

    **else if** *uncertainty* > 0 and $\mu .Hamming\_dist\le {\theta }_{{h}^{\prime}}$ and *μ*. *slide*_*name* not in *V* **then**

     *R*_*ret*_. *insert*(*μ*)

     *V*. *insert*(*μ*. *slide*_*name*)

 *R*_*ret*_ ← SORTING(*R*_*ret*_)

 **return** *R*_*ret*_[0: *K*]

Clean (Algorithm 6) aims to remove outliers and the patches that are less similar to the query in *R*_*I*_. It takes summaries of patch *S*_*m*_ and *S*_*l*_ from the previous stage as input, removing *r* whose result length ∣*r*∣ is less than the *O*_*l*_ or greater than the *O*_*h*_ quantiles. Additionally, we take the average of the mean Hamming distances in the top ${k}_{{\theta }_{h}^{\prime}}$ patches for each *r* ∈ *R*_*I*_ as a threshold denoted by ${\theta }_{{h}^{\prime}}$, using this to filter out *r* whose mean Hamming distance in top ${k}_{{\theta }_{h}^{\prime}}$ retrieval is greater than ${\theta }_{{h}^{\prime}}$. After cleaning the results, we sort them on the basis of the uncertainty calculated from Weighted-Uncertainty-Cal in ascending order.

**Algorithm 5 Uncertainty Calculation**

 **function** Weighted-Uncertainty-Cal(*r*_*i*_, *D*_*inv*)

 *label*_*cnt*, *dist* ← {}, {}

 **for** *pos*_*index*, *μ* in *r*_*i*_ **do**

  $$label\_cnt[\mu .diagnosis]\leftarrow label\_cnt[\mu .diagnosis]+D\_inv[\mu .diagnosis]\times \frac{1}{pos\_index}$$

  *dist*. *insert*(*μ*.*hamming*_*dist*)

 **for** *lb*, *cnt* in *label*_*cnt* **do**

  **if** *cnt* < 1 **then**

   *label*_*cnt*[*lb*] ← 1

 *Ent* = Entropy(*label*_*cnt*)

 **return** *Ent*, *label*_*cnt*, *dist*

**Algorithm 6 Results Cleaning**

 **function** Clean(*S*_*m*_, *S*_*l*_)

 //*S*_*m*_:An array that stores tuples composed of the index *i*, the entropy, the Hamming distance of all patches, and the total number of patches for each *r*_*i*_ in *R*_*I*_.

 //*S*_*l*_:An array that stores the total number of patches for each *r*_*i*_ in *R*_*I*_.

 *tmp* ← {}

 $${k}_{{\theta }_{h}^{\prime}}\leftarrow 5$$

 *O*_*l*_ ← 5%

 *O*_*h*_ ← 95%

 *l* ← 3

 // When the unique results length is less than 3, we keep the original *S*_*m*_.

 **if** Unique(*S*_*l*_)≥*l* **then**

  **for** *res* in *S*_*m*_ **do**

   **if** *res*. *r*. *len*()≤Quantile(*S*_*l*_, *O*_*l*_) or *res*. *r*. *len*()≥Quantile(*S*_*l*_, *O*_*h*_) **then**

    del res

   **else**

    *tmp*. *insert*(Mean $(res.dist[0:{k}_{{\theta }_{h}^{\prime}}])$)

 **else**

  **for** *res* in *S*_*m*_ **do**

   *tmp*. *insert*(Mean $(res.dist[0:{k}_{{\theta }_{h}^{\prime}}])$)

 $${\theta }_{{h}^{\prime}}\leftarrow$$ Mean(*tmp*)

 **for** *res* in *S*_*m*_ **do**

  **if** Mean $(res.dist[0:m]) > {\theta }_{h}^{\prime}$ **then**

   del *res*

 *S*_*m*_ ← Sort-Ascending(*S*_*m*_, *key* = *Ent*)

 **return** (*S*_*m*_)

We can now return the slide from *r*_*i*_ at the beginning of the sorted *S*_*m*_ on the basis of the uncertainty. However, a drawback of this approach is that the low uncertainty of the first several *r* ∈ *S*_*m*_ could be caused by the domination of the most frequent diagnoses in the given anatomic site. For example, the most frequent occurrences of the top 5 entries in *S*_*m*_ could be KIRC, BLCA, KIRP, KIRP and KIRP for the urinary site. In this case, the query slide would be diagnosed as KIRP on the basis of the majority vote. Therefore, the first and second entries that dominate the urinary site cases should not be considered during retrieval. We leverage the Filtered-By-Prediction (Algorithm 7) to mitigate this issue. This function takes the summation of the diagnosis occurrences from the top *k*_*f*_ certain elements in *S*_*m*_. It first uses the diagnosis with the maximum score as a pseudo-ground truth diagnosis from the top *k*_*f*_ most certain elements. Afterwards, it removes all elements whose maximum occurrence diagnosis disagrees with the pseudo-ground truth. To return final results *R*_ret,*I*_ of slide query *I*, we take the slide name and its diagnosis in *r*_*i*_ pointed to by *S*_*m*_ one by one. If the uncertainty of *r*_*i*_ is zero, we take all (*p*_*i*_, *μ*_*i*_). Otherwise, we use ${\theta }_{{h}^{\prime}}$ again to ignore (*p*_*i*_, *μ*_*i*_) whose Hamming distance is greater than the threshold. We sort *R*_ret,*I*_ first by uncertainty in ascending order then by Hamming distance in descending order if the uncertainty is a tie.

#### Training details of VQ-VAE

We randomly sampled 20 1,024 × 1,024 patches at ×20 magnification from 9,286 diagnostic slides across 29 TCGA projects. All patches were converted from RGB to Pytorch tensors, then normalized such that all values lie within $\left[-1,1\right]$. The model was trained using the Adam optimizer with a learning rate of 10^−3^ without weight decay and without AMSgrad. We used default settings for other hyperparameters in Adam (*β*_1_=0.9 and *β*_1_=0.999, and *ϵ* = 10^−8^). We trained our model with a batch size of 4 for 10 epochs. We applied gradient clipping techniques by setting the gradient threshold to 1.0. The hyperparameter *α* in VQ-VAE was also set to 1 (equation 2). In the patch speed experiments, we followed the same training receipts above, except that we trained on a patch of size 256 × 256.

### Ablation study

We conducted two ablation studies: one to show that using a pretrained VQ-VAE does not confer an advantage when compared with the Yottixel pipeline and a second to test the benefit of each function in our ranking module. For the first study, we conducted a top-5 nearest neighbour search on Kather100k by using the VQ-VAE integer index and the Yottixel index. For the ranking module, we compared the performance of the following four settings: (1) Naive: removing Clean and Filtered-By-Prediction and treating each diagnosis occurrence in the mosaic retrieval result equally (that is, replacing the assignment in line 4 in Algorithm 5 with 1). (2) +Weighed count: applying Uncertainty-Cal to the ranking module only. (3) +Clean: applying Uncertainty-Cal and Clean to the ranking module. (4) +Filter: applying all functions to the ranking module.

**Algorithm 7 Results Filtering by Prediction**

 **function** Filtered_By_Prediction(*S*_*m*_, *S*_*lb*_)

 //*S*_*m*_: An array that stores tuples composed of the index *i*, the entropy, the Hamming distance of all patches, and the total number of patches for each *r*_*i*_ in *R*_*I*_.

 //*S*_*lb*_: A nested hash table that stores the index of *r* in *R*_*I*_ as the key and its weighted diagnosis occurrences table as value.

 *cnt* ← {}

 *k*_*f*_ ← 5

 **for** *s*_*m*_ in *S*_*m*_[0:*k*_*f*_] **do**

  // Calculate the score of each diagnosis

  **for** *d* in *S*_*lb*_[*s*_*m*_. *i*] **do**

   *cnt*[*d*] ← *cnt*[*d*] + *S*_*lb*_[*s*_*m*_. *i*][*d*]

 *plb*_*list* ← Sort-Descending(*cnt*)

 *p* ← 0

 // A while loop is used here to avoid the case that the plb remove all *s*_*m*_.

 **while** **do**

  *plb* ← *plb*_*list*[*p*]

  *removed* ← {}

  **for** *s*_*m*_ in *S*_*m*_[0: *k*_*f*_] **do**

   *pred* ← Max(*S*_*lb*_[*s*_*m*_. *i*])

   **if** *pred* ≠ *plb* **then**

    *removed*. *insert*(*s*_*m*_. *i*)

  **if** *removed*. *len*() ≠ *k*_*f*_ **then**

   break

  **else**

   *p* ← *p* + 1

 **return** *removed*

### Visualization

We build confusion matrices for each site using each slide diagnosis as ground truth along the *y* axis and Mv(ret[: *k*]) as predicted diagnosis along the *x* axis. For the Hamming distance matrix, we inspect the Hamming distance between the query slide and each result in ret[: *k*] one by one, adding the Hamming distance to the associated diagnosis label and infinity to others. The infinity here is defined as Hamming distance threshold *θ*_*h*_ plus 1, as *θ*_*h*_ is the maximum distance we can have in our pipeline. The final Hamming distance matrix is obtained by dividing the total number of slides in the given anatomic site.

### Evaluation metrics

For all experiments, we remove the slide with the same patient identification as the query slide in the database (that is, leave-one-patient-out evaluation). Since the ability to diagnose each subtype of cancer is the paramount task in search-based disease classification^53^ and a false-negative case does harm to the patient’s health, we evaluate the accuracy of each subtype by dividing the number of correct prediction by the total number of subtype in the site. The prediction of a query is determined by the majority vote of slide label in the top-*k* retrieval (that is, MV@k). Therefore, the overall mean accuracy mMV@k for a given subtype *L* is defined as:

9 $${{{\rm{mMV@k}}}}=\frac{1}{Q}\mathop{\sum }\limits_{i=1}^{Q}{\mathbb{1}}[L={{{\rm{Mv}}}}({{{{\rm{ret}}}}}_{i}[:k])]\,,$$

where *Q* is the number of slides for the given cancer subtype and MV(ret_*i*_[: *k*]) is the predicted diagnosis of *S*_*i*_ taken from the majority vote of the top-*k* retrieval ret_*i*_. We use *k* = 5 for disease subtyping and *k* = 10 for anatomic site prediction for comparative analysis with ref. ^35^. Similar to the related works that focused on top retrieval^38,54^ (that is, *k* ≤ 5), we also report *k* = 1 and *k* = 3 to evaluate the performance of the top hit. In addition, we use the definition of mAP@k below to provide another perspective.

10 $${{{\rm{AvP@k}}}}=\frac{\mathop{\sum }\nolimits_{i = 1}^{k}{{{{\rm{Prec}}}}}_{i}\cdot {{{{\rm{Rel}}}}}_{i}}{k},$$

11 $${{{\rm{mAP@k}}}}=\frac{{{{\rm{AvP@k}}}}}{| Q| }$$

where AvP@k, Prec_*i*_ and Rel_*i*_ respectively denote average precision up to *k*, precision at *i* and relevant indicator at *i* (that is, 1 if the item at the position *i* is relevant and 0 otherwise). For AvP@k, we adapted the original definition^55^ by changing the denominator to the maximum number of slides whose labels match the query’s. We did this adaption to ensure that the mAP@k in each subtype has the same scale. We used *k* = 5 for the disease subtyping task.

### Computational hardware and software

We stored all WSIs, patches, segmentation masks and mosaics across multiple disks with total size around 27TB. Segmentation, patching, mosaic extraction and search of WSIs were performed on a CPU (AMD Ryzen Threadripper 3970X 32-Core Processor). The VQ-VAE pretraining and feature extraction were performed on 4 NVIDIA 2080Ti GPU. The whole SISH pipeline was written in Python (version 3.7.0), with the following external package: h5py (2.10.0), matplotlib(3.3.0), numpy (1.19.1), opencv-python (4.3.0.38), pillow (7.2.0), pandas (1.1.0), scikit-learn (0.23.1), seaborn (0.10), scikit-image (0.17.2), torchvision (0.6.0), tensorboard (2.3.0) and tqdm (4.48.0). We used Pytorch (1.5.0) for deep learning. All plots were created by matplotlib (version 3.2.2) and seaborn (version 0.10.1). The pie charts were created using Adobe Illustrator.

### WSI datasets

There are three datasets in our slide-level retrieval experiment: diagnostic slides in The Cancer Genome Atlas (TCGA), Clinical Proteomic Tumor Analysis Consortium (CPTAC) and BWH in-house data.

#### TCGA and CPTAC diagnostic slides

We downloaded all diagnostic slides from the TCGA genomic data commons and the CPTAC websites. To fairly compare with Yottixel, we used slides from the same 13 anatomic sites for anatomic site retrieval and the same 29 diagnoses for disease subtype retrieval in the TCGA. There are 503 CPTAC-Clear Cell Renal Cell Carcinoma slides from 216 patients, 544 CPTAC-UCEC slides from 240 patients, 679 CPTAC-LUSC slides from 210 patients, 669 CPTAC-LUAD slides from 224 patients and 283 CPTAC-SKCM slides from 93 patients. All slides were processed at ×20. Detailed slide and patient numbers are reported in Supplementary Table 4.

#### In-house diagnostic slides

From the WSI database at Brigham and Women’s Hospital, we collected 8,035 diagnostic WSIs that span 9 anatomic sites with 37 primary cancer subtypes. Further details of the dataset are available in Supplementary Table 5. All slides were processed at ×20.

### Patch datasets

There are five datasets in the patch retrieval experiments: colon tissues (Kather100k), BWH prostate, lung tissues (WSSS4LUAD), atlas of digital pathology (Atlas) and breast tissues (BCSS). We provide details for each dataset below.

#### Kather100k^45^

The data contain 100,000 224 × 224 tissue patches from colon at ×20 without colour normalization, from the National Center for Tumor Diseases (NCT) biobank and the University Medical Center Mannheim (UMM). The tissue patches are adipose (ADI), background (BACK), debris (DEB), lymphocytes (LYM), mucus (MUC), smooth muscle (MUS), normal colon mucosa (NORM), cancer-associated stroma (STR) and colorectal adenocarcinoma epithelium (TUM).

#### BWH in-house prostate

In this cohort, each whole-slide image is from a different patient. For the prostate data used in patch-level retrieval, we collected 23 slides at ×20, scanned the slides using Hamamatsu S210 and annotated regions in each slide by gleason score 3, 4, 5 or normal, where the number of patches for each category is 2,355, 2,289, 171 and 2,000, respectively. Detailed slide and patient numbers are reported in Supplementary Table 5.

#### WSSSS4LUAD^46^

We used the training part of the data that contains 10,091 annotated patches from 49 WSIs from the Guangdong Provincial People’s Hospital (GDPH) and 14 WSIs from lung adenocarinoma in TCGA. Each patch ranges from 150 to 300 × 150 to 300 pixels and has one annotation from [’stroma’, ’stroma + tumour’, ’tumour’, ’normal’]. We only considered the patch with annotation stroma, tumour and normal, which resulted in 4,698 patches. The slides were scanned using Leica and Aperio-AT2 at ×10.

#### Atlas^47^

This dataset contains 17,668 272 × 272 patches from 100 glass slides scanned by Huron TissueScope LE1.2 in a private cohort mentioned in ref. ^47^ with 38 histology tissue types annotation organized in 3 hierarchies. The patches were cropped from 100 slides at ×40 from various organs. We only considered the label in the first hierarchy which are: epithelial (E), connective proper (C), blood (H), skeletal (S), adipose (A), muscular (M), nervous (N), glandular (G) and transport vessel (T). Multiple labels for a patch is common in this dataset.

#### BCSS^48^

This dataset contains the pixel-level annotation from 151 TCGA-breast WSI at ×40 and are grouped into five categories: tumour, stroma, inflammatory, necrosis and other. Specifically, the tumour class includes the predominant tumour, ductal carcinoma in situ (DCIS) and angioinvasion, and the inflammatory infiltrates include plasma cell, lymphocytes and other infiltrates. We only considered the first four classes since they are more clinically relevant. We cropped each patch into 256 × 256 and assigned the patch with all labels appearing in the region. The number of total patches is 7,691. Having multiple labels for a patch is possible in this dataset. We considered the search correct if most of the categories in the search result match one of the categories in the query patch.

### WSI processing

#### Segmentation

We used the automatic segmentation tool in clustering-constrained attention multiple instance learning^9^ (CLAM) to generate the segmentation mask for each slide. The tool first applies a binary threshold to a downsampled whole-slide image on the hue, saturation and value colour space to generate a binary mask and then refines the mask by median blurring and morphological closing to remove the artefacts. After getting the approximate contours of the tissue, the tool filters out the tissue contours and cavities on the basis of a certain area threshold.

#### Patching

After segmentation, we cropped the contours into 1,024 × 1,024 patches without overlapping at ×20. For ×40 whole slides, we first cropped them into 2,048 × 2,048 patches and then downsampled them to 1,024 × 1,024 to get the equivalent patches at ×20.

#### Mosaic generation

We followed the mosaic generation process proposed in the Yottixel paper^36^. The algorithm first applies *K*-mean clustering to the RGB features extracted from each patch, with number of cluster *K* = 9. Within each cluster, we run *K*-means clustering again on the coordinate of each patch by setting the number of clusters to 5% of the cluster size. If the number of clusters is <1 in the second stage, we took all coordinates within that cluster. Except for the number of clusters, we used all default settings in Scikit-learn for *K*-means clustering. To get better quality of mosaics, we collected 101 patches for both debris/pen smudges and tissue to train a logistic regression on the basis of local binary pattern histogram feature to remove the unmeaningful regions. We used the default setting from the Scikit-learn package in logistic regression and used the rotate invariant binary pattern from Scikit-image package with *P* = 8 and *R* = 1. The bin number of the histogram was set to 128.

#### Artefacts removal

Rarely, we found that the generated mosaic might contain patches with nearly completely white background. We removed such patches from the mosaic if the white regions accounted for over 90% of pixels in each patch. We applied the binary threshold method in OpenCV with a threshold value of 235 to determine the area (in number of pixels) of white regions.

### Ethics oversight

The retrospective analysis of archival pathology slides was approved by the Mass General Brigham (MGB) IRB office under protocol 2020P000233.

### Reporting summary

Further information on research design is available in the Nature Research Reporting Summary linked to this article.

## Supplementary information

Main Supplementary Information Supplementary figures and tables.

Reporting Summary

Data Supplementary Tables 7–16.

## Source data

Source Data Fig. 3 Source data.

Source Data Fig. 4 Source data.

Source Data Fig. 5 Source data.

Source Data Fig. 6 Source data.

Source Data Fig. 7 Source data.

## Data Availability

The TCGA diagnostic whole-slide data and corresponding labels are available from the National Institutes of Health (NIH) genomic data commons (https://portal.gdc.cancer.gov). The CPTAC whole-slide data and the corresponding labels are available from the NIH cancer imaging archive (https://cancerimagingarchive.net/datascope/cptac). Supplementary Table 19 provides access links to publicly available patch-level datasets. Source data for the figures are provided with this paper. All reasonable requests for academic use of in-house raw and analysed in-house data can be addressed to the corresponding author. All requests will be promptly reviewed to determine whether the request is subject to any intellectual property or patient-confidentiality obligations, will be processed in concordance with institutional and departmental guidelines, and will require a material transfer agreement.

## Code availability

All scripts, checkpoints, preprocessed mosaics and pre-built database to reproduce the findings are available at https://github.com/mahmoodlab/SISH. We implemented all our methods in Python and used Pytorch as the primary package for training VQ-VAE. The code base is made available for non-commercial and academic purposes.
