# Supplementary material for: Fast and scalable search of whole-slide images via self-supervised deep learning
Source: Nat Biomed Eng. 2022 Oct 10;6(12):1420–34. doi: 10.1038/s41551-022-00929-8 (PMC9792371; doi:10.1038/s41551-022-00929-8)
Supplement: Supplementary file 1 — Supplementary figures and tables. [file 41551_2022_929_MOESM1_ESM.pdf]

nature portfolio

https://doi.org/10.1038/s41551-022-00929-8

Supplementary information

Fast and scalable search of whole-slide images via self-supervised deep learning

In the format provided by the authors and unedited

Contents

Supplementary Figures		Page
**Figure 1**	Confusion matrices and hamming distance matrices in TCGA cohort.	2
**Figure 2**	Confusion matrices and hamming distance matrices in BWH cohort.	3
**Figure 3**	The majority-5 accuracy on Kather100k dataset.	4
**Figure 4**	VQ-VAE model architecture	4
**Figure 5**	Example of encoding a whole slide image	5
**Figure 6**	Results for interpretation study	5
**Figure 7**	Results for cross independent cohort retrieval	6

Supplementary Tables		Page
**Table 1**	Numerical results of SISH and Yottixel on TCGA	7
**Table 2**	Numerical results of SISH and Yottixel on BWH cohort	7
**Table 3**	Numerical results of SISH and Yottixel on rare type cancers	9
**Table 4**	Summary of public data	10
**Table 5**	Summary of in-house BWH data	11
**Table 6**	5-fold cross validation accuracy of CLAM for each disease on BWH urinary	12
**Table 7**	Individual retrieval result on TCGA cohort.	12
**Table 8**	Individual retrieval result on TCGA plus CPTAC cohort.	12
**Table 9**	Individual retrieval result on BWH independent test cohort	12
**Table 10**	Individual retrieval results on rare diseases	12
**Table 11**	Individual retrieval result on anatomic site retrieval on TCGA cohort	12
**Table 12**	Individual retrieval result on Kather100k	12
**Table 13**	Individual retrieval result on BWH prostate	13
**Table 14**	Individual retrieval result on WSSS4LUAD	13
**Table 15**	Individual retrieval result on Atlas	13
**Table 16**	Individual retrieval result on BCSS	13
**Table 17**	Summary of comparison with other methods	13
**Table 18**	Summary of symbols used in Methods	14
**Table 19**	Summary of public datasets and the access links	15

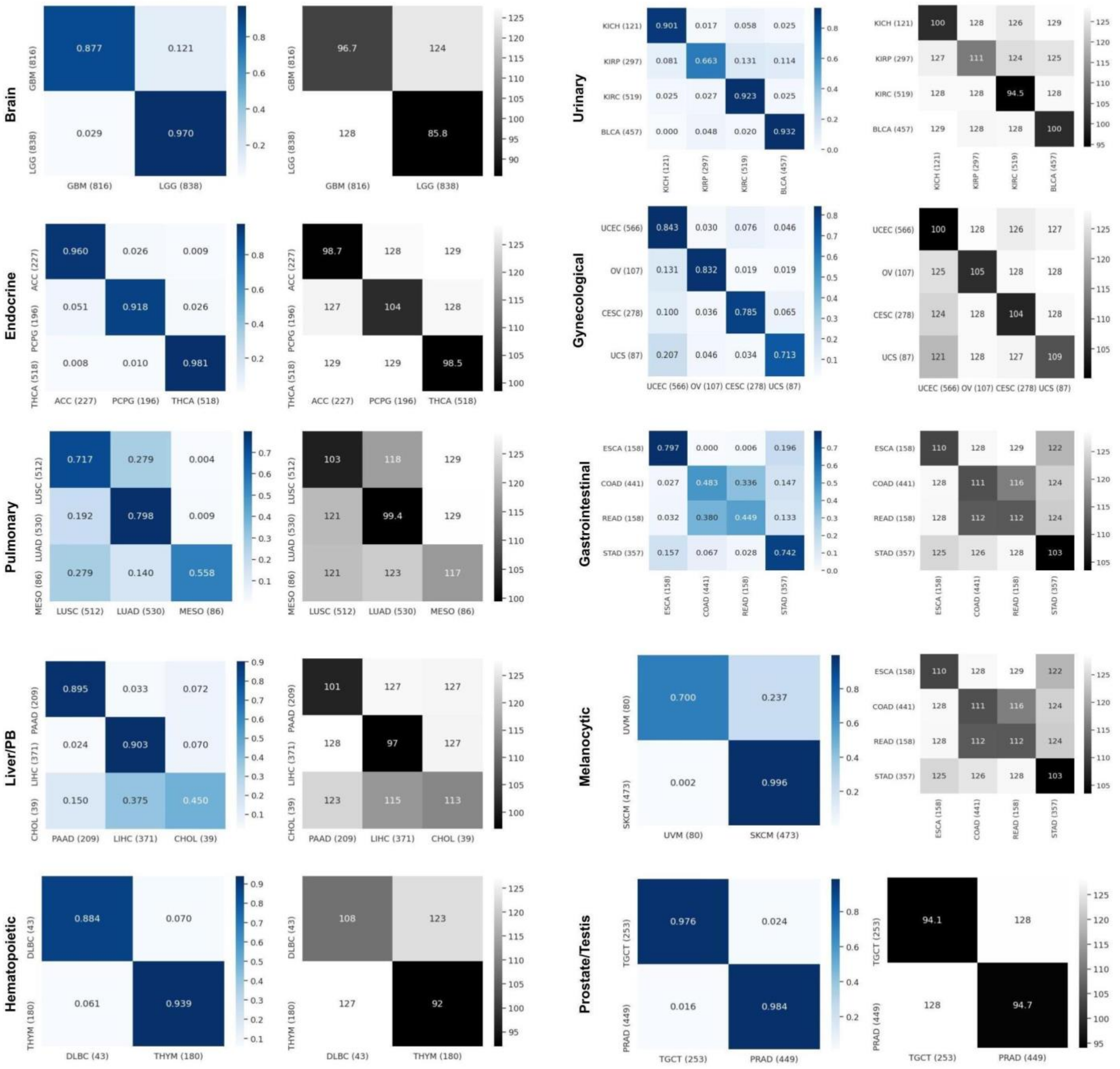

Supplementary Figure 1. Confusion matrices and hamming distance matrices in TCGA cohort. The confusion matrices and hamming distance matrices of SISH on TCGA cohort. A dark diagonal line, which suggested the majority results SISH retrieves match the queried diagnosis. The hamming distance matrices further explained the trends in the confusion matrices. The dark diagonal line shows the smallest hamming distance values in all sites, demonstrating that slides with different diagnosis are pushed far away in the hamming distance space. The x-axis and y-axis correspond to the model prediction and the ground truth respectively.

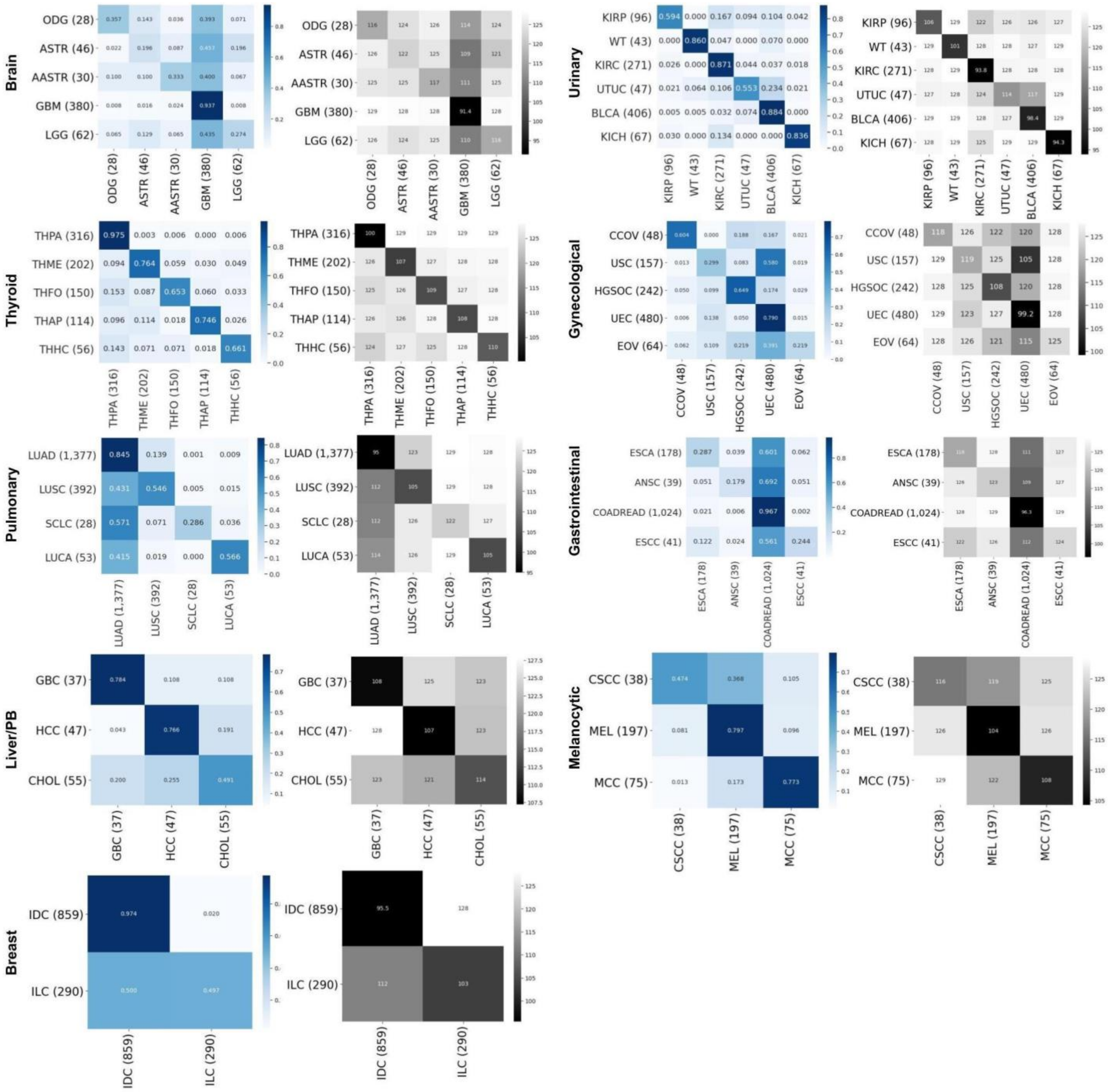

Supplementary Figure 2. Confusion matrices and hamming distance matrices in BWH cohort. The confusion matrices and hamming distance matrices of SISH on BWH cohort. A dark diagonal line, which suggested the majority results SISH retrieves match the queried diagnosis. The hamming distance matrices further explained the trends in the confusion matrices. The dark diagonal line shows the smallest hamming distance values in all sites, demonstrating that slides with different diagnosis are pushed far away in the hamming distance space. The x-axis and y-axis correspond to the model prediction and the ground truth respectively.

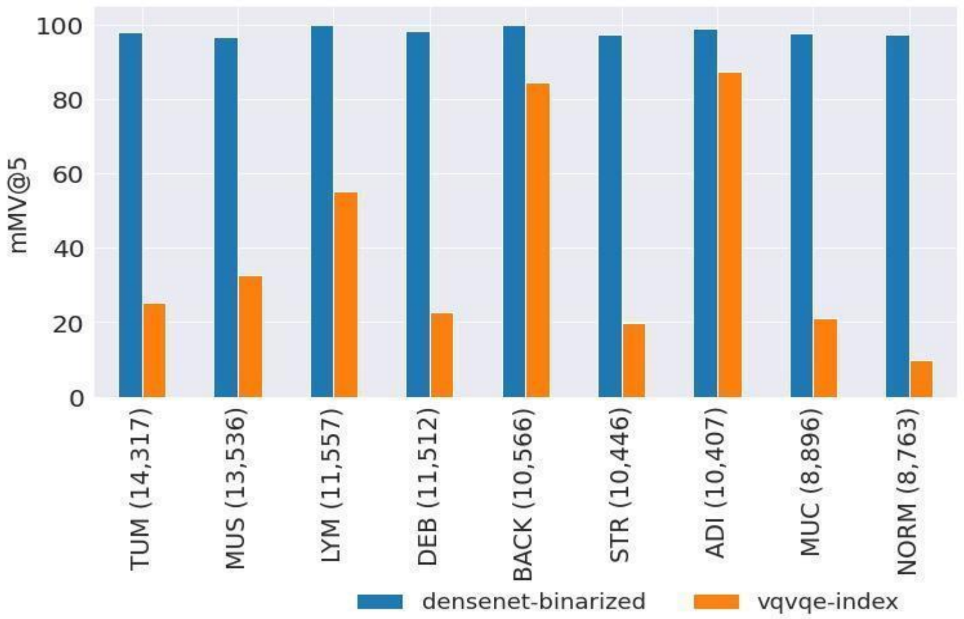

Supplementary Figure 3. The majority-5 accuracy on Kather100k dataset. The comparison of 5-nearest neighbor classification results by using VQ-VAE integer index and DenseNet feature. VQ-VAE integer index are generally worse, which means the pretrained on dataset does not lead to unfair comparison. To clarify, our method is a hierarchical search where the first level search relies on VQ-VAE index and the second level relies on Densenet feature. The benefit VQ-VAE index is in the first stage where it makes the number of neighbors needed to visit a constant. Detail results can be found in **Speed and Interpetability** section.

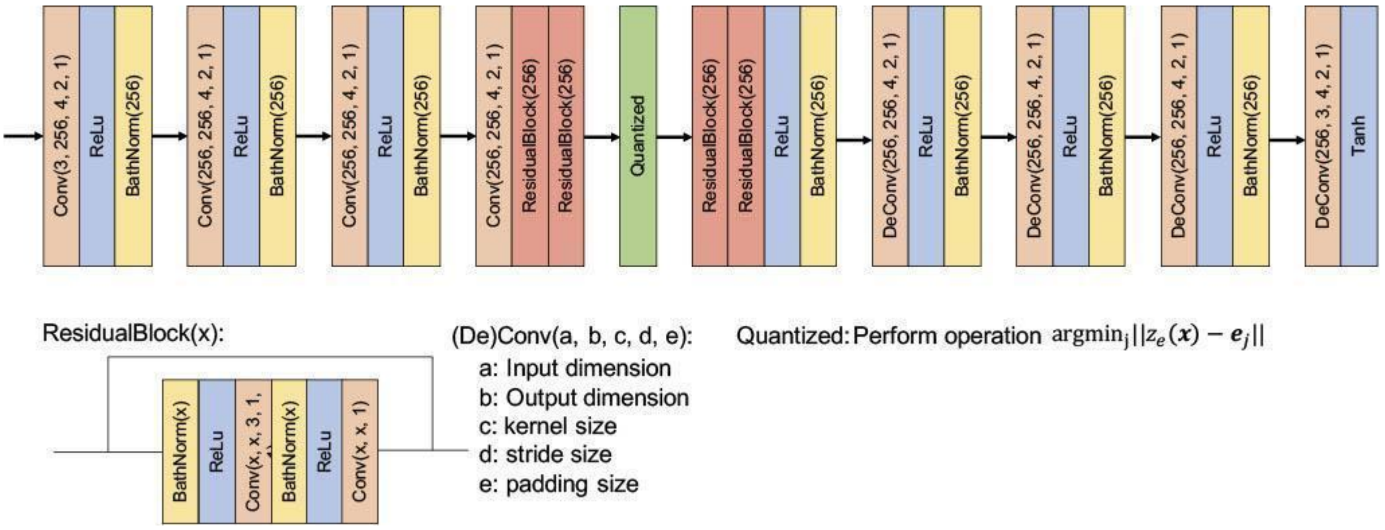

Supplementary Figure 4. VQ-VAE model architecture. The VQ-VAE architecture we used in our pipeline. The modules before the Quantized layer are encoder made up by a sequence of convolution, relu activation, batch normalization and residual module. After quantized layer is a decoder made up by a sequence of residual module, deconvolution, batch normalization and relu activation. We applied a tanh activation for the output layer

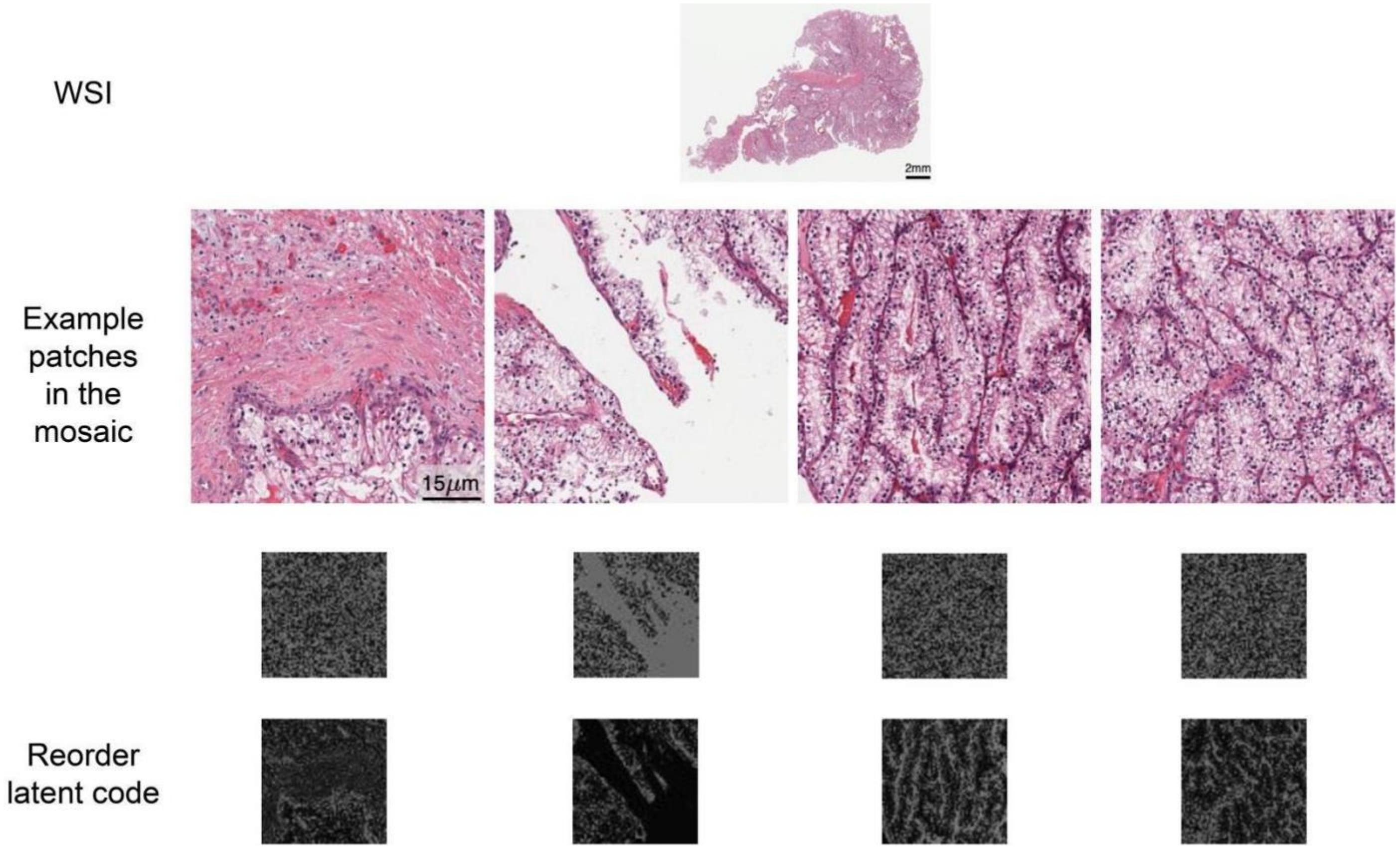

Supplementary Figure 5. Example of encoding a whole slide image. An example of how we convert a WSI to mosaics and reordered latent codes through mosaic generation, VQ-VAE encoding and reorder the latent code. Note that the reordered latent codes contain more semantic information in the original mosaics.

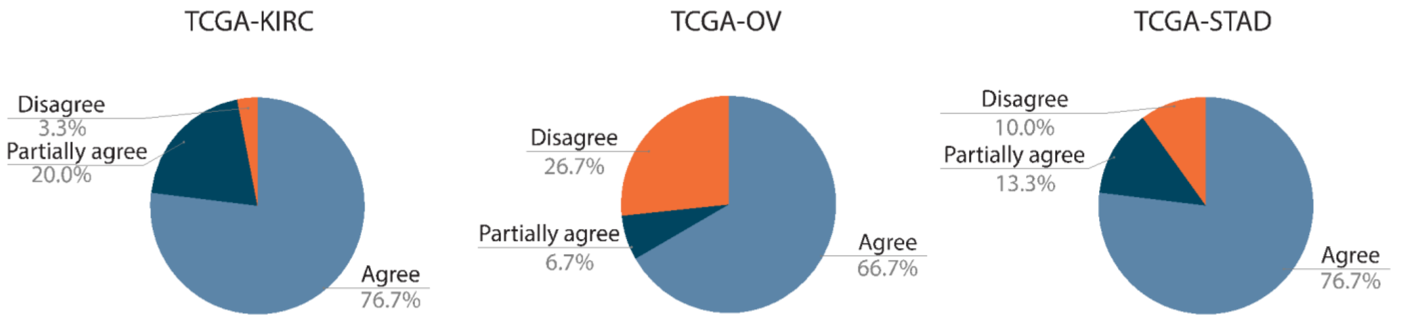

Supplementary Figure 6. Results for interpretation study. We studied kidney renal clear cell carcinoma (KIRC), Ovarian serous cycstadenocarcinoma (OV) and Stomach adenocarcinoma (STAD) in TCGA cohort with 99.29%, 83.18% and 74.23% mMV@5 score, respectively. Each study contains 30 randomly selected queries and each query contain 1 to 5 ROIs. A pathologist rate `agree`, `partially agree`, and `disagree` based on whether the region contains tumor. The proportion of `agree` plus `partially agree` exceeds over 70% in all studies.

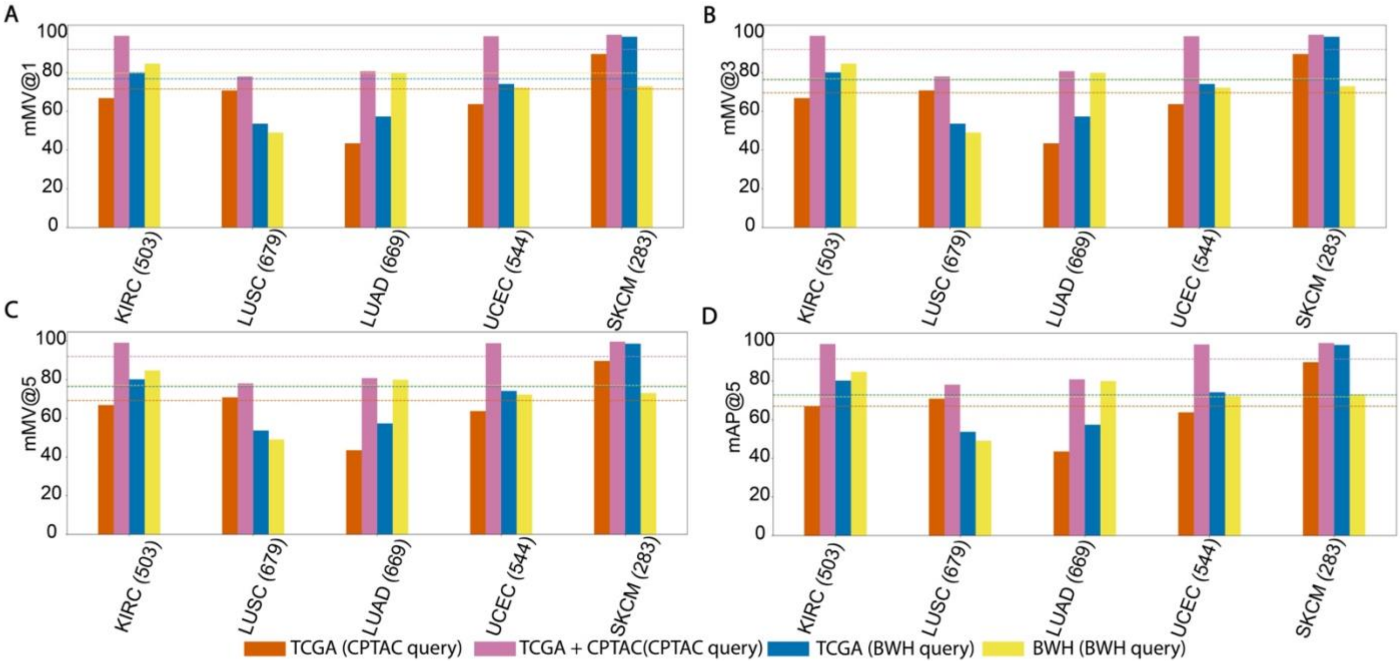

Supplementary Figure 7. Results for cross independent cohort retrieval. (A)-(D): The mMV@1,3,5, and mAP@5 results of cross independent cohort retrieval. The database was constructed on TCGA, TCGA + CPTAC, and BWH. The corresponding query slide is from CPTAC and BWH.

	mMV@1		mMV@3		mMV@5	
	SISH	Yottixel	SISH	Yottixel	SISH	Yottixel
**Brain**						
LGG	**97.02**	42.50	**97.14**	66.14	**97.02**	89.77
GBM	**87.62**	43.00	**87.75**	67.09	87.75	**91.18**
**Endocrine**						
ACC	**95.59**	42.5	**96.04**	68.165	**96.04**	93.83
PCPG	**91.84**	46.25	**91.84**	67.51	**91.84**	88.77
THCA	**98.07**	50.0	**98.26**	73.8	**98.07**	97.66
**Liver**						
LIHC	**85.71**	43.5	**90.30**	68.575	90.30	**93.65**
PAAD	**85.17**	42.5	**88.04**	66.77	89.47	**91.04**
CHOL	**58.97**	22.5	**56.41**	33.04	**46.15**	43.58
**Gastrointestinal**						
COAD	**40.82**	32.50	45.58	**54.32**	48.30	**76.14**
ESCA	**81.01**	30.00	**81.01**	44.94	**79.75**	59.87
READ	**51.90**	5.00	**46.84**	7.60	**44.94**	10.19
STAD	**72.55**	33.50	**73.11**	54.31	74.23	**75.12**
**Prostate**						
TGCT	**97.64**	50.00	**97.64**	74.61	97.64	**99.21**
PRAD	**98.44**	50.00	**98.44**	74.22	**98.44**	98.43
**Hematopoietic**						
DLBC	**90.70**	30.00	**88.37**	44.07	**88.37**	58.13
THYM	**93.89**	50.00	**94.44**	74.44	93.89	**98.87**
**Urinary**						
KIRP	**67.00**	30.00	**66.33**	48.61	66.33	**67.22**
KIRC	**91.52**	45.00	**92.10**	68.33	**92.29**	91.66
KICH	**90.08**	35.00	**90.08**	55.46	**90.10**	75.92
BLCA	**91.90**	46.00	**92.78**	70.91	93.22	**95.81**
**Melanocyte**						
UVM	**77.50**	42.50	**72.50**	63.13	70.00	**83.75**
SKCM	**99.58**	50.00	**99.58**	74.79	**99.58**	99.57
**Pulmonary**						
LUSC	**71.68**	40.00	**71.48**	60.85	71.68	**81.70**
LUAD	**80.38**	32.50	**79.81**	51.73	**79.81**	70.96
MESO	**66.28**	5.00	**62.79**	6.57	**55.81**	8.13
**Gynecology**						
OV	**85.05**	32.50	**84.11**	49.74	**83.18**	66.98
UCS	**74.71**	20.00	**72.41**	31.11	**71.26**	42.22
CESC	**82.01**	30.00	**80.58**	46.23	**78.78**	62.45
UCEC	**82.33**	45.00	**82.86**	68.61	84.28	**92.22**
**Average**	**82.31**	36.80	**82.02**	56.40	**81.33**	76.00

Supplementary Table 1. Numerical results of SISH and Yottixel on TCGA. mMV@1,3,5 scores for each subtype on TCGA. The numeric results of Yottixel were estimated from the figure in the original paper.

	Subtype	mMV@1		mMV@3		mMV@5		mAP@5	
		SISH	Yottixel	SISH	Yottixel	SISH	Yottixel	SISH	Yottixel
**Brain**	GBM	87.11	**91.84**	90.26	**96.32**	93.68	**97.63**	**88.98**	88.22
	LGG, NOS	**45.16**	40.32	32.26	**37.10**	27.42	**33.87**	**21.85**	20.49
	ASTR	**23.91**	17.39	15.21	**17.39**	**19.56**	13.04	**10.97**	8.90
	AASTR	**50.00**	33.33	**33.33**	30.00	**33.33**	20.00	**23.99**	14.42
	ODG	**53.57**	32.14	**46.43**	35.71	**35.71**	25.00	**26.19**	17.76
**Thyroid**	THPA	**96.20**	86.08	**96.84**	95.25	97.47	**98.73**	**95.36**	79.73
	THAP	**77.19**	71.93	**76.32**	69.30	**74.56**	65.79	**69.61**	53.68
	THFO	68.00	**72.00**	**65.33**	64.67	**65.33**	64.67	**59.26**	49.61
	THME	77.72	**80.69**	77.23	**82.67**	76.73	**83.66**	**74.48**	72.51
	THHC	**71.43**	64.29	**69.64**	62.50	**66.07**	51.79	**50.78**	38.53
**Gastrointesinal**	COAD READ	92.09	**92.97**	95.41	**96.78**	96.68	**98.14**	**92.85**	89.86
	ESCA	42.13	**47.19**	35.39	**41.01**	28.65	**44.38**	24.91	**30.20**
	ESCC	**26.83**	14.63	**21.95**	14.63	**24.39**	7.32	**12.38**	6.81
	ANSC	**38.46**	33.33	17.95	**30.77**	17.95	**25.64**	**15.16**	13.26
**Gynecological**	UEC	73.54	**80.83**	77.29	**86.88**	78.96	**89.58**	**72.26**	68.99
	HGSOC	**60.33**	52.07	**62.81**	53.72	**64.88**	57.02	**50.24**	34.77
	USC	**42.04**	31.21	**39.49**	26.11	**29.94**	24.84	**24.27**	18.55
	EOV	**35.94**	20.31	**28.15**	14.06	**21.88**	18.75	**15.93**	11.93
	CCOV	**64.58**	35.42	**62.50**	37.50	**60.42**	29.17	**37.62**	16.89
**Liver biliary**	HCC	**74.47**	**74.47**	76.60	**78.72**	76.60	**82.98**	**67.50**	52.61
	GBC	**72.97**	56.76	**72.97**	62.16	**78.38**	62.16	**64.24**	49.05
	CHOL	49.09	**69.09**	49.09	**74.54**	49.09	**69.09**	46.09	**46.19**
**Cutaneous**	MEL	74.11	**76.65**	79.19	**84.77**	79.70	**88.83**	**73.00**	66.80
	MCC	**82.67**	69.33	**80.00**	66.67	**77.33**	61.33	**72.90**	52.89
	CSCC	**63.16**	34.21	**50.00**	28.95	**47.37**	28.95	**42.18**	21.45
**Pulmonary**	LUAD	79.74	**84.97**	83.44	**92.30**	84.46	**94.19**	**79.90**	77.25
	LUSC	**59.95**	49.49	**57.65**	44.39	**54.59**	44.64	**49.05**	35.87
	LUCA	**73.58**	20.75	**60.38**	13.21	**56.60**	9.43	**45.51**	8.31
	SCLC	**42.86**	17.86	**35.71**	17.86	**28.57**	10.71	**23.79**	12.57
**Urinary**	BLCA	86.45	**91.38**	87.68	**92.12**	88.42	**94.58**	**86.81**	86.67
	KIRC	85.61	**89.30**	86.72	**92.62**	87.08	**95.57**	**84.69**	80.72
	KICH	**85.07**	70.15	**86.57**	64.18	**83.58**	64.18	**81.56**	57.22
	KIRP	**61.46**	58.33	**60.42**	54.17	**59.38**	50.00	**51.89**	39.99
	WT	**88.37**	76.74	**88.37**	72.09	**86.05**	76.74	**82.22**	67.36
	UTUC	**63.83**	42.55	**55.32**	25.53	**55.32**	27.66	**32.55**	17.97
**Breast**	IDC	**97.56**	93.36	97.44	**98.25**	97.44	**99.19**	**97.30**	88.17
	ILC	50.34	**53.10**	**50.69**	50.00	**49.66**	46.90	48.56	**49.30**
**Average**		**65.34**	57.47	**62.22**	56.89	**60.90**	55.57	**53.97**	44.47

Supplementary Table 2. Numerical results of SISH and Yottixel on BWH cohort. SISH achieved 5-8% higher mMV@k (k=1,3,5) than Yottixel and outperformed 33 out of 37 subtypes on mAP@5.

	Subtype	mMV@1		mMV@3		mMV@5		mAP@5	
		SISH	Yottixel	SISH	Yottixel	SISH	Yottixel	SISH	Yottixel
Brain	ASTR	**34.78**	26.09	**36.96**	26.09	32.61	**36.96**	**24.55**	19.92
	AASTR	43.33	**53.33**	**43.33**	**43.33**	**40.00**	**40.00**	**29.31**	21.96
	ODG	46.43	**60.71**	39.29	**53.57**	**42.86**	**42.86**	**27.33**	26.26
	PAST	**65.00**	45.00	**55.00**	40.00	**55.00**	45.00	**38.68**	27.48
	AODG	14.29	**21.43**	**14.29**	**14.29**	**7.14**	**7.14**	6.57	**9.74**
Thyroid	THAP	**80.70**	76.32	**78.95**	72.81	**77.19**	71.93	**73.98**	57.46
	THFO	**76.67**	74.67	**74.00**	69.33	**72.67**	70.66	**68.09**	53.09
	THME	78.22	**83.66**	78.71	**87.62**	80.20	**89.11**	**77.59**	75.33
	THHC	**82.14**	69.64	**80.36**	66.07	**75.00**	55.36	**59.30**	41.20
Gastrointestinal	ESCC	**58.54**	**58.54**	58.54	**70.73**	53.66	**65.85**	**52.54**	45.87
	ANSC	**64.10**	61.54	64.10	**66.67**	66.67	**71.79**	**63.53**	51.48
Gynecological	USC	**92.99**	91.08	94.27	**96.18**	93.63	**98.09**	**93.04**	84.83
	EOV	**46.88**	**46.88**	45.31	**53.13**	43.75	**50.00**	**38.79**	35.47
	CCOV	**62.50**	47.92	**60.42**	47.92	**58.33**	50.00	**54.85**	32.21
Liver biliary	PAAD	91.87	**92.82**	**94.74**	**94.74**	94.26	**95.69**	**91.41**	81.77
	CHOL	70.21	**71.28**	69.15	**71.28**	69.15	**74.47**	**63.12**	57.52
	PANET	**80.52**	71.43	**80.52**	76.62	**76.62**	75.32	**70.55**	53.37
	GBC	**78.38**	40.54	**78.38**	40.54	**70.27**	37.84	**51.73**	28.87
Pulmonary	LUCA	**96.23**	83.02	**96.23**	94.34	**96.23**	**96.23**	**95.78**	78.58
	SCLC	**64.29**	**64.29**	60.71	**67.86**	57.14	**67.86**	**52.61**	48.98
Urinary	KICH	97.34	**98.40**	97.34	**98.94**	97.34	**98.94**	**97.39**	97.33
	WT	**95.35**	83.72	**95.35**	86.04	**95.35**	90.70	**92.84**	78.75
	UTUC	**85.11**	78.72	85.11	**87.23**	**85.11**	82.98	**80.45**	70.45
Average		**69.82**	65.26	**68.74**	66.32	**66.96**	65.86	**61.03**	51.21

Supplementary Table 3. Numerical results of SISH and Yottixel on rare type cancers. SISH achieved 1-5% higher mMV@k (k=1,3,5) than Yottixel and outperformed 22 out of 23 subtypes on mAP@5.

Diagnosis	#WSIs	#Patients	source
GBM	816	378	TCGA
LGG	838	490	TCGA
ACC	227	56	TCGA
PCPG	196	176	TCGA
THCA	518	505	TCGA
COAD	441	433	TCGA
ESCA	158	156	TCGA
READ	158	157	TCGA
STAD	357	332	TCGA
UCEC	566	505	TCGA
CESC	278	268	TCGA
UCS	87	53	TCGA
OV	107	106	TCGA
DLBC	43	43	TCGA
THYM	180	121	TCGA
UVM	80	80	TCGA
SKCM	473	431	TCGA
CHOL	39	39	TCGA
LIHC	371	363	TCGA
PAAD	209	183	TCGA
LUAD	530	467	TCGA
MESO	86	74	TCGA
LUSC	512	478	TCGA
BLCA	457	386	TCGA
KIRC	519	513	TCGA
KICH	121	109	TCGA
KIRP	297	273	TCGA
TGCT	254	149	TCGA
PRAD	449	403	TCGA
BRCA	1,123	1,054	TCGA
HNSCC	471	449	TCGA
Mesenchymal	600	254	TCGA
C-KIRC	503	216	CPTAC
C-UCEC	544	240	CPTAC
C-SKCM	283	93	CPTAC
C-LUSC	679	210	CPTAC
C-LUAD	669	224	CPTAC
**Total**	**14,239**	**10,467**	

Supplementary Table 4. Summary of public data. Slide and patient number of public cohorts used in the experiment. We merge all diagnosis in the Breast, Head and Neck and Mesenchymal since we only use site information for the experiment.

Diagnosis	Oncotree	#WSIs	#Patients
Glioblastoma Multiforme	GBM	380	380
Low-Grade Glioma, NOS	LGG	62	62
Astrocytoma	ASTR	46	46
Anaplastic Astrocytoma	AASTR	30	30
Oligodendroglioma	ODG	28	28
Pilocytic Astrocytoma	PAST	20	20
Anaplastic Oligodendroglioma	AODG	14	14
Papillary Thyroid Cancer	THPA	316	316
Medullary Thyroid Cancer	THME	202	202
Follicular Thyroid Cancer	THFO	150	150
Anaplastic Thyroid Cancer	THAP	114	114
Hurthle Cell Thyroid Cancer	THHC	56	56
Colorectal Adenocarcinoma	COADREAD	1,024	1,024
Esophageal Adenocarcinoma	ESCA	178	178
Esophageal Squamous Cell Carcinoma	ESCC	41	41
Anal Squamous Cell Carcninoma	ANSC	39	39
Uterine Endometrioid Carcinoma	UEC	480	480
High-Grade Serous Ovarian Cancer	HGSOC	242	242
Uterine Papillary Serous Carcinoma	USC	157	157
Endometrioid Ovarian Cancer	EOV	64	64
Clear Cell Ovarian Cancer	CCOV	48	48
Cholangiocarcinoma	CHOL	55	55
Hepatocellular Carcinoma	HCC	47	47
Gallbladder Cancer	GBC	37	37
Pancreatic Neuroendocrine Tumor	PANET	77	77
Melanoma	MEL	197	197
Merkel Cell Carcinoma	MCC	75	75
Cutaneous Squamous Cell Carcinoma	CSCC	38	38
Lung Adenocarcinoma	LUAD	1,377	1,377
Lung Squamous Cell Carcinoma	LUSC	392	392
Lung Carcinoid	LUCA	53	53
Small Cell Lung Cancer	SCLC	28	28
Bladder Urothelial Carcinoma	BLCA	406	406
Kidney Renal Clear Cell Carcinoma	KIRC	271	271
Kidney Renal Papillary Cell Carcinoma	KIRP	96	96
Kidney Chromophobe	KICH	67	67
Upper Tract Urothelial Carcinoma	UTUC	47	47
Wilms Tumor	WT	43	43
Breast Invasive Ductal Carcinoma	IDC	859	859
Breast Invasive Lobular Carcinoma	ILC	290	290
Prostate (patch level experiment)		23	23
Total		8,169	8,169

Supplementary Table 5. Summary of in-house BWH data. The number of slides and patients in the BWH cohort with oncotree code for subtype in WSI subtyping experiments.

	KICH (67)	WT (43)	BLCA (406)	UTUC (47)
CLAM				
Fold 0	94.44	66.67	87.80	70.00
Fold 1	100	100	85.37	30.00
Fold 2	100	88.89	89.02	40.00
Fold 3	88.99	88.89	76.83	60.00
Fold 4	100	44.44	91.46	50.00
Mean±std.	96.66±4.97	77.78±22.22	86.10±5.63	50.00±15.81
SISH	83.58	86.05	88.42	55.32

Supplementary Table 6. 5-fold cross validation accuracy of CLAM for each disease on BWH urinary. Supervised model on the case with few data tend to have high variance which makes it difficult to evaluate.

Supplementary Table 7. Individual retrieval result on TCGA cohort. The individual retrieval result of SISH for each anatomic site on TCGA (n = 9, 367). Each row represents a query slide and the top-5 results with the corresponding hamming distance.

This large table is available in the accompanying excel sheet.

Supplementary Table 8. Individual retrieval result on TCGA plus CPTAC cohort. The individual retrieval result of SISH for each anatomic site on TCGA plus CPTAC (n = 6, 791). Each row represents a query slide and the top-5 results with the corresponding hamming distance.

This large table is available in the accompanying excel sheet.

Supplementary Table 9. Individual retrieval result on BWH independent test cohort. The individual retrieval result of SISH for each anatomic site on BWH test cohort (n = 8, 035). Each row represents a query slide and the top-5 result with the corresponding hamming distance.

This large table is available in the accompanying excel sheet.

Supplementary Table 10. Individual retrieval result on rare diseases. The individual retrieval result of SISH for each anatomic site on rare diseases (n = 1, 785). Each row represents a query slide and the top-5 result with the corresponding hamming distance.

This large table is available in the accompanying excel sheet.

Supplementary Table 11. Individual retrieval result on anatomic site retrieval on TCGA cohort. The individual retrieval result of SISH for each anatomic site on TCGA (n = 11, 561). Each row represents a query slide and the top-10 results with the corresponding hamming distance

This large table is available in the accompanying excel sheet.

Supplementary Table 12. Individual retrieval result on Kather100k The individual retrieval result of SISH for Kather100k (n = 100, 000). Each row represents a query slide and the top-5 result with the corresponding hamming distance.

This large table is available in the accompanying excel sheet.

Supplementary Table 13. Individual retrieval result on BWH prostate. The individual retrieval result of SISH for BWH prostate (n = 6, 779). Each row represents a query slide and the top-5 result with the corresponding hamming distance.

This large table is available in the accompanying excel sheet.

Supplementary Table 14. Individual retrieval result on WSSS4LUAD. The individual retrieval result of SISH for WSSS4LUAD (n = 4, 693). Each row represents a query slide and the top-5 result with the corresponding hamming distance.

This large table is available in the accompanying excel sheet.

Supplementary Table 15. Individual retrieval result on Atlas. The individual retrieval result of SISH for Atlas (n = 17, 668). Each row represents a query slide and the top-5 result with the corresponding hamming distance.

This large table is available in the accompanying excel sheet.

Supplementary Table 16. Individual retrieval result on BCSS. The individual retrieval result of SISH for Atlas (n = 7, 691). Each row represents a query slide and the top-5 result with the corresponding hamming distance.

Methods	Patch query	WSI query	No need for patch level annotation	Search time complexity
Hedge et al (2019)	√			$O(log(n))$
Karla et al (2019)	√	√	√	$O(nlog(n))$
Zheng et al (2019)	√			$O(n)$
SISH	√	√	√	$O(1)$

Supplementary Table 17. The summary of several recent related works. We reported each related work and their ability and limitation. The column ”No need for patch level annotation” refers to whether patch level annotation needed when WSI used as a query

Symbol	Definition
$\boldsymbol{x}$	Input vector of VQ-VAE.
$\boldsymbol{e}$	Latent vector in the codebook.
$\boldsymbol{z_{i,1}}, \boldsymbol{z_{i,2}}, \boldsymbol{z_{i,3}}$	Original latent codes from VQ-VAE for the input patch i
$\boldsymbol{K}$	The number of codewords in the codebook.
$\boldsymbol{D}$	The dimension of each codeword in the codebook
$\boldsymbol{M}$	A constant that determines the complexity of our pipeline
$\boldsymbol{I}$	Whole slide image
C	Width of interval used in index expansion ($C = 50 \times 10^{11}$)*
$\boldsymbol{T}$	Number of times an index expands itself for one side ($T = 10$)*
$\boldsymbol{N}$	A constant that used to ensure the weighted count in WEIGHTED- UNCERTAINTY-CAL for all diagnoses are close to 1
$\boldsymbol{m_i}$	Integer index of the patch i in the query mosaic
$\boldsymbol{h_i}$	Binarized texture feature from DenseNet121
$\boldsymbol{m_{i,c+}}$	Expanded integer index for patch i (left-side)
$\boldsymbol{m_{i,c-}}$	Expanded integer index for patch i (right-side)
$\boldsymbol{\theta_h}$	Maximum threshold of the hamming distance between two patches to be considered similar*
$\boldsymbol{k_{succ}}$	Number of times to perform forward search ($k_{succ} = 375$)*
$\boldsymbol{k_{pred}}$	Number of times to perform backward search ($k_{pred} = 375$)*
$\boldsymbol{R_I}$	Collection of search results r_i of whole slide image I
$\boldsymbol{S_m}$	A list stores tuple of form (index of r_i, entropy, hamming distance info in μ_{ij} , length of r_i)
$\boldsymbol{S_l}$	An array that only stores the length of r_i
$\boldsymbol{S_{lb}}$	A nested dictionary that stores the disease occurrences in r_i
$\boldsymbol{O_l}$	The lower percentile to be considered in the sample in S_l ($O_l = 5\%$)*
$\boldsymbol{O_h}$	The upper percentile to be considered in the sample in S_l ($O_h = 95\%$)*
l	The number of unique length of patch's retrieval results in the query mosaic (*l*=3)*
$\boldsymbol{r_i}$	Search result for each patch i in the query mosaic
$\boldsymbol{p}$	Integer index of a patch
$\boldsymbol{p_{ij}}$	j -th integer index in the i-th search result r_i
$\boldsymbol{\mu}$	Metadata associated with a patch in the database, including the slide-level label, slide name, slide extension and position where the patch from
$\boldsymbol{\mu_{ij}}$	Metadata associated with the$j$ -th patch in i -th search result r_i, which includes all information from μ plus the hamming distance between h_i and h_j
$\boldsymbol{k_{\theta'_h}}$	Number of patches used to calculate the threshold θ'_h($k_{\theta'_h} = 5$)*
$\boldsymbol{k_f}$	The first k_f element used to produce pseudo label in FILTER-BY-PREDICTION ($k_f = 5$)*
$\boldsymbol{\theta'_h}$	Maximum threshold of the hamming distance to filter out S_m and determine the similarity during extraction

Supplementary Table 18: The symbols and definitions used in the online method. The value of the symbols marked with * were determined by the results across all experiments. We only choose a set of parameter that works best for all experiment except θ_h. One can further optimize for each anatomic site accordingly. For θ_h, we set it to 128 for all slide level experiment and kather100k, 150 for other patch data retrieval and 256 for patch speed experiment.

Dataset	Link
TCGA data	https://portal.gdc.cancer.gov/repository
CPTAC data	https://www.cancerimagingarchive.net/datascope/cptac/
Kather100k	https://zenodo.org/record/1214456#.Ysde5hNKj-Y
Atlas	https://www.dsp.utoronto.ca/projects/ADP/
BCSS	https://bcsegmentation.grand-challenge.org/
WSSS4LUAD	https://wsss4luad.grand-challenge.org/

Supplementary Table 19: Access link to public datasets used in the study.
