# Supplementary material for: Fast and scalable search of whole-slide images via self-supervised deep learning
Source: Nat Biomed Eng. 2022 Oct 10;6(12):1420–34. doi: 10.1038/s41551-022-00929-8 (PMC9792371; doi:10.1038/s41551-022-00929-8)
Supplement: Supplementary file 2 — Reporting Summary [file 41551_2022_929_MOESM2_ESM.pdf]

nature portfolio

Corresponding author(s): Faisal Mahmood

Last updated by author(s): Jul 13, 2022

Reporting Summary

Nature Portfolio wishes to improve the reproducibility of the work that we publish. This form provides structure for consistency and transparency in reporting. For further information on Nature Portfolio policies, see our Editorial Policies and the Editorial Policy Checklist.

Statistics

For all statistical analyses, confirm that the following items are present in the figure legend, table legend, main text, or Methods section.

n/a	Confirmed	
☐	☒	The exact sample size (n) for each experimental group/condition, given as a discrete number and unit of measurement
☒	☐	A statement on whether measurements were taken from distinct samples or whether the same sample was measured repeatedly
☒	☐	The statistical test(s) used AND whether they are one- or two-sided<br>*Only common tests should be described solely by name; describe more complex techniques in the Methods section.*
☒	☐	A description of all covariates tested
☒	☐	A description of any assumptions or corrections, such as tests of normality and adjustment for multiple comparisons
☐	☒	A full description of the statistical parameters including central tendency (e.g. means) or other basic estimates (e.g. regression coefficient) AND variation (e.g. standard deviation) or associated estimates of uncertainty (e.g. confidence intervals)
☒	☐	For null hypothesis testing, the test statistic (e.g. F, t, r) with confidence intervals, effect sizes, degrees of freedom and P value noted<br>*Give P values as exact values whenever suitable.*
☒	☐	For Bayesian analysis, information on the choice of priors and Markov chain Monte Carlo settings
☒	☐	For hierarchical and complex designs, identification of the appropriate level for tests and full reporting of outcomes
☒	☐	Estimates of effect sizes (e.g. Cohen's d, Pearson's r), indicating how they were calculated

Our web collection on statistics for biologists contains articles on many of the points above.

Software and code

Policy information about availability of computer code

Data collection	All in-house slides were scanned by Aperio and Hamamatsu S210, and were accessed through openslide (3.4,1). Code for data and image processing was implemented in Python (3.7.0), and is available at https://github.com/mahmoodlab/SISH.
Data analysis	The implementation of the pipeline for model development and evaluation are available from https://github.com/mahmoodlab/SISH. All codes were written in Python (3.7.0) and use Pytorch (1.5.0) for deep learning. These additional Python libraries were used: h5py (2.10.0), matplotlib(3.3.0), numpy (1.19.1), opencv-python (4.3.0.38), pillow (7.2.0), pandas (1.1.0), scikit-learn (0.23.1), seaborn (0.10),scikit-image (0.17.2), torchvision (0.6.0) tensorboard (2.3.0) and tqdm (4.48.0). We used Adobe Illustrator to create pie charts and to arrange all figures.

For manuscripts utilizing custom algorithms or software that are central to the research but not yet described in published literature, software must be made available to editors and reviewers. We strongly encourage code deposition in a community repository (e.g. GitHub). See the Nature Portfolio guidelines for submitting code & software for further information.

Data

Policy information about availability of data

All manuscripts must include a data availability statement. This statement should provide the following information, where applicable:

- Accession codes, unique identifiers, or web links for publicly available datasets
- A description of any restrictions on data availability
- For clinical datasets or third party data, please ensure that the statement adheres to our policy

The TCGA diagnostic whole-slide data and corresponding labels are available from the National Institutes of Health (NIH) genomic data commons (https://portal.gdc.cancer.gov). The CPTAC whole-slide data and the corresponding labels are available from the NIH cancer imaging archive (https://cancerimagingarchive.net/datascope/cptac). Supplementary Table 19 provides access links to publicly available patch-level datasets. All reasonable requests for

academic use of in-house raw and analysed in-house data can be addressed to the corresponding author. All requests will be promptly reviewed to determine whether the request is subject to any intellectual property or patient-confidentiality obligations, will be processed in concordance with institutional and departmental guidelines, and will require a material transfer agreement.

Field-specific reporting

Please select the one below that is the best fit for your research. If you are not sure, read the appropriate sections before making your selection.

☒ Life sciences ☐ Behavioural & social sciences ☐ Ecological, evolutionary & environmental sciences

For a reference copy of the document with all sections, see nature.com/documents/nr-reporting-summary-flat.pdf

Life sciences study design

All studies must disclose on these points even when the disclosure is negative.

Sample size	No statistical tests were used to determine the sample size. We used all available data for model development.<br><br>Public datasets: After excluding poor-quality slides, we used 11,561 slides from the TCGA and 2,678 slides from the CPTAC. For the kather100k, BCSS and Atlas, we use all data in the released datasets. For WSSS4LUAD, we used the data in the challenge training set with stroma, tumour and normal annotation (see Methods for details).<br><br>BWH independent test cohort: We used all available whole-slide images from our in-house repository corresponding to rare and common diagnoses. Based on the availability of slides and after excluding poor-quality slides, we used a total of 8,169 in house slides.
Data exclusions	Pre-established exclusion criteria include slides with significant marking covering the tissue area, damaged slides and missing slides. Slides with markings that do not predominantly cover tissue regions were not excluded.
Replication	We provide the data and instructions to reproduce the results at https://github.com/mahmoodlab/SISH. Replication was successful when all requirements were met.
Randomization	No patient recruitment was performed for this study, and randomization was not necessary.
Blinding	Blinding was not needed for this retrospective image-analysis study.

Reporting for specific materials, systems and methods

We require information from authors about some types of materials, experimental systems and methods used in many studies. Here, indicate whether each material, system or method listed is relevant to your study. If you are not sure if a list item applies to your research, read the appropriate section before selecting a response.

Materials & experimental systems

n/a	Involved in the study	
☒	☐	Antibodies
☒	☐	Eukaryotic cell lines
☒	☐	Palaeontology and archaeology
☒	☐	Animals and other organisms
☐	☒	Human research participants
☒	☐	Clinical data
☒	☐	Dual use research of concern

Methods

n/a	Involved in the study	
☒	☐	ChIP-seq
☒	☐	Flow cytometry
☒	☐	MRI-based neuroimaging

Human research participants

Policy information about studies involving human research participants

Population characteristics	Public Data: TCGA,CPTAC, Kather100k, BCSS, WSSS4LUAD and Atlas contain data from a diverse population representing multiple hospitals.<br><br>In-house data: All patient cases between 2002–2020 were queried from the pathology database.
Recruitment	No patient recruitment was necessary for the use of histology whole-slide images retrospectively.
Ethics oversight	The Mass General Brigham IRB committee approved the retrospective image analysis of pathology slides.

Note that full information on the approval of the study protocol must also be provided in the manuscript.
